# Volumetric assessment of extracranial carotid artery aneurysms

**DOI:** 10.1038/s41598-019-44553-0

**Published:** 2019-05-30

**Authors:** E. E. de Vries, C. J. H. C. M. van Laarhoven, H. J. Kuijf, C. E. V. B. Hazenberg, J. A. van Herwaarden, M. A. Viergever, G. J. de Borst

**Affiliations:** 1 Department of Vascular Surgery, University Medical Center Utrecht, University Utrecht, PO Box 85500, Utrecht, The Netherlands; 2 Image Sciences Institute, University Medical Center Utrecht, University Utrecht, PO Box 85500, Utrecht, the Netherlands

**Keywords:** Preclinical research, Experimental models of disease

## Abstract

The extracranial carotid artery aneurysm (ECAA) is a rare pathology for which clinical treatment guidelines are lacking. In general, symptoms or growth of the aneurysm sac are thought to indicate intervention. ECAAs may present in a large variety of shapes and sizes, and conventional diameter measurements fail to indicate geometrical differences. Therefore, we propose a protocol to measure ECAA size by 3D volumetric assessment. The volumes of 40 ECAAs in computed tomography angiography (CTA) images were measured through manual segmentation, by two independent operators. Volumes of the entire internal carotid artery (ICA) and the ECAA were measured separately. Excellent inter- and intraoperator reliability was found for both ICA and ECAA volumes, with all intraclass correlation coefficients above 0.94. Bland-Altman analysis revealed normal differences for both inter- and intraoperator agreement. For all volumes, similarity of the segmentations was excellent. Outliers were explained by presence of intraluminal ECAA thrombus, which hampered identification of the aneurysm outer wall. These results implicate robustness of our protocol, which is designed as a step-up towards (semi)automatic volumetric measurements to monitor patients with ECAA. Future (semi)automatic volumetric assessments are recommended and such techniques can be developed and validated using the proposed protocol and manual reference segmentations.

## Introduction

The extracranial carotid artery aneurysm (ECAA) is a rare vascular pathology, of which the natural clinical course, and risk factors for adverse outcome are largely unknown^1,2^. As a consequence, currently no evidence-based guidelines exist on treatment of ECAAs. Asymptomatic, non-growing aneurysms are generally treated conservatively^3^, while surgical or endovascular aneurysm exclusion may be considered in case of symptomatic or growing ECAA^2^. However, a standardized method to measure ECAA size or growth is currently not available.

At present, ECAA size is mostly defined by determination of maximum diameter. These diameters can be assessed by measuring the bi-directional maximum aneurysm diameter from outer to outer vessel wall, extracted from the well-established approach for abdominal aortic aneurysms (AAAs)^4–6^. However, ECAAs may present in a large variety of shapes and sizes. The dilatation of ECAA may be focal and saccular, or extensive and fusiform, or a combination of both, and maximum aneurysm diameters between 4 and 60 mm have been reported^3,7,8^. Furthermore, it is imaginable that ECAA growth might occur in other directions than just obliquely. Thus, assessment of geometrical differences by comparing maximum aneurysm diameters is inadequate as we lack the appropriate imaging tools. A standardized protocol to measure 3D ECAA volume - instead of 2D diameters - would theoretically be able to reliably determine aneurysm size and growth. These measures could then be used for follow-up of ECAA patients (and ultimately for patients with any type of aneurysm), for pre-treatment monitoring, and underpinning of treatment decisions.

Therefore, the aim of this study was to develop and evaluate a robust protocol for volume measurement of ECAAs.

## Methods

### Participants

Twenty patients with an ECAA located in the internal carotid artery (ICA) gave informed consent and were included from our Carotid Aneurysm Registry (www.carotidaneurysmregistry.com). Ethical approval for this study (12/020761) was provided by the Medical Research Ethics Committee of University Medical Center Utrecht, Utrecht, The Netherlands on 12 June 2012, and all research was conducted according to the principles of the Declaration of Helsinki (59th amendment, Seoul 2008) and in accordance with the Dutch Medical Research Involving Human Subjects Act (WMO). The registry protocol has been published elsewhere^9^. Briefly, any patient aged 18-years or older with an ECAA is included in this ongoing registry, independent of etiology or treatment strategy. Patients who, or their legal representative, are unable or unwilling to sign the informed consent were excluded. Selected patients for the current study had two computed tomography angiography (CTA) scans of the carotids made at different time points. Hence, in total 40 scans were included. The CTAs had been performed for evaluation or treatment planning of ECAAs between 2008 and 2017, in the University Medical Center Utrecht. As specified within the registry protocol^9^, fusiform aneurysms were defined as ≥150% diameter increase of the non-affected ICA diameter, while saccular aneurysms were defined as a distended sac of any size affecting only part of the ICA circumference.

### Volume measurement protocol

The protocol for volume measurement of ECAAs was developed by an expert panel, which consisted of three vascular surgeons (G.B., J.H., C.H.) and two dedicated researchers (E.V., C.L.). Each case was measured in a twofold manner. First, the volume of the entire ICA (including ECAA) was measured, from carotid bifurcation up until the entrance of the ICA in the skull base (method 1). Hereafter, the ECAA volume was measured separately (method 2).

#### Entire ICA (method 1)

**Figure 1:**
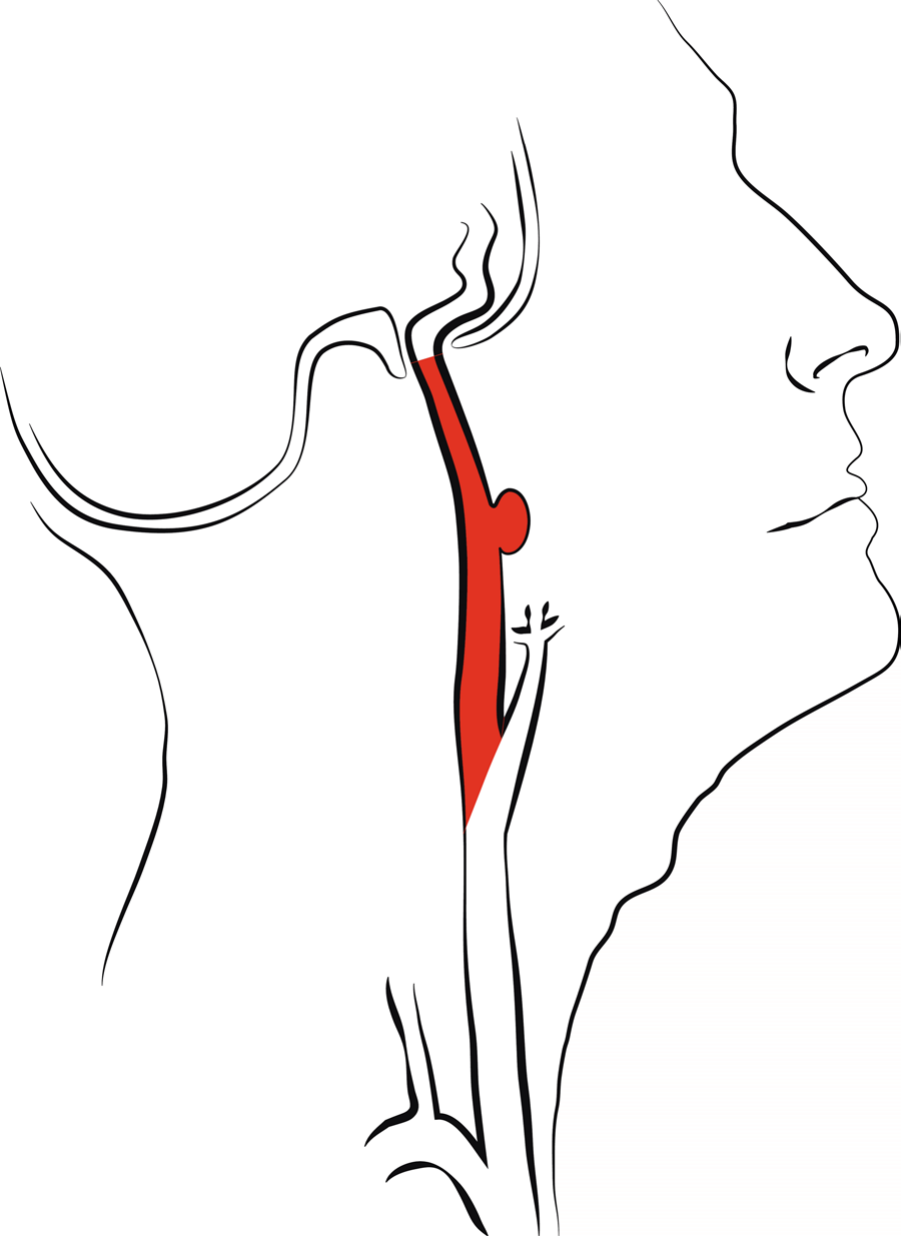
Measurement method 1: Entire ICA.

The starting point of the ICA was the carotid bifurcation (Fig. 1). On sagittal and coronal views, the ipsilateral common and external carotid arteries were segmented, and subtracted from the segmented proximal ICA on axial view. Distally, the ICA was segmented up until entrance in the skull base, defined as the first slice which showed at least 50% of the ICA surrounded by bone.

#### Solely the ECAA (method 2)

Since ECAAs come in different shapes, a different approach was inevitable to define the borders of saccular versus fusiform aneurysms.

**Figure 2:**
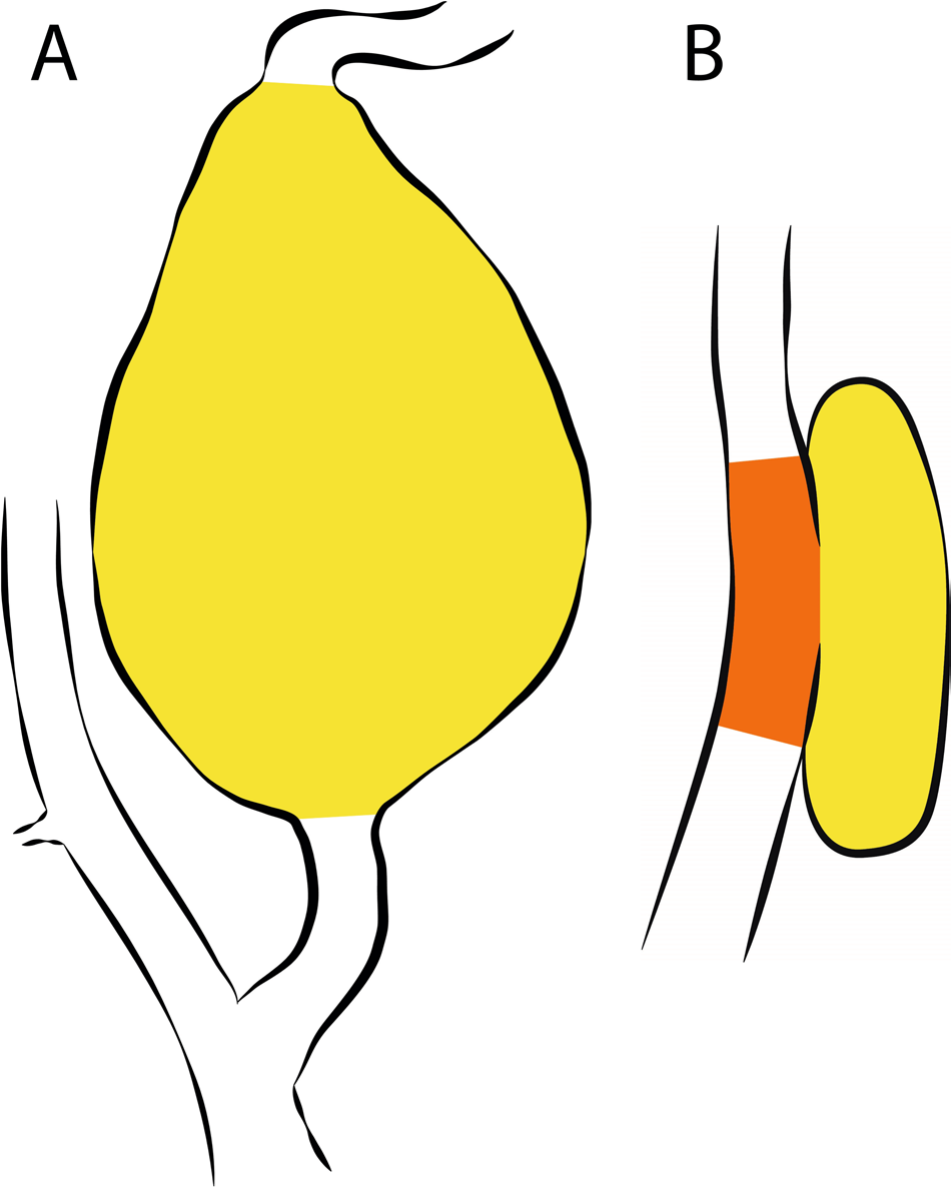
Measurement method 2: Solely the ECAA. (**A**) Fusiform aneurysm. (**B**) Saccular aneurysm: measurement of solely the aneurysm sac (yellow), and vessel adjacent to aneurysm sac (orange).

Fusiform aneurysms: The aneurysm was segmented from the clear starting point of vessel dilatation onwards (immediately ≥150%; Fig. 2A). If the widening of the vessel commenced gradually, segmentation started when the vessel had widened with ≥125% compared with the healthy ipsilateral ICA.

**Figure 3:**
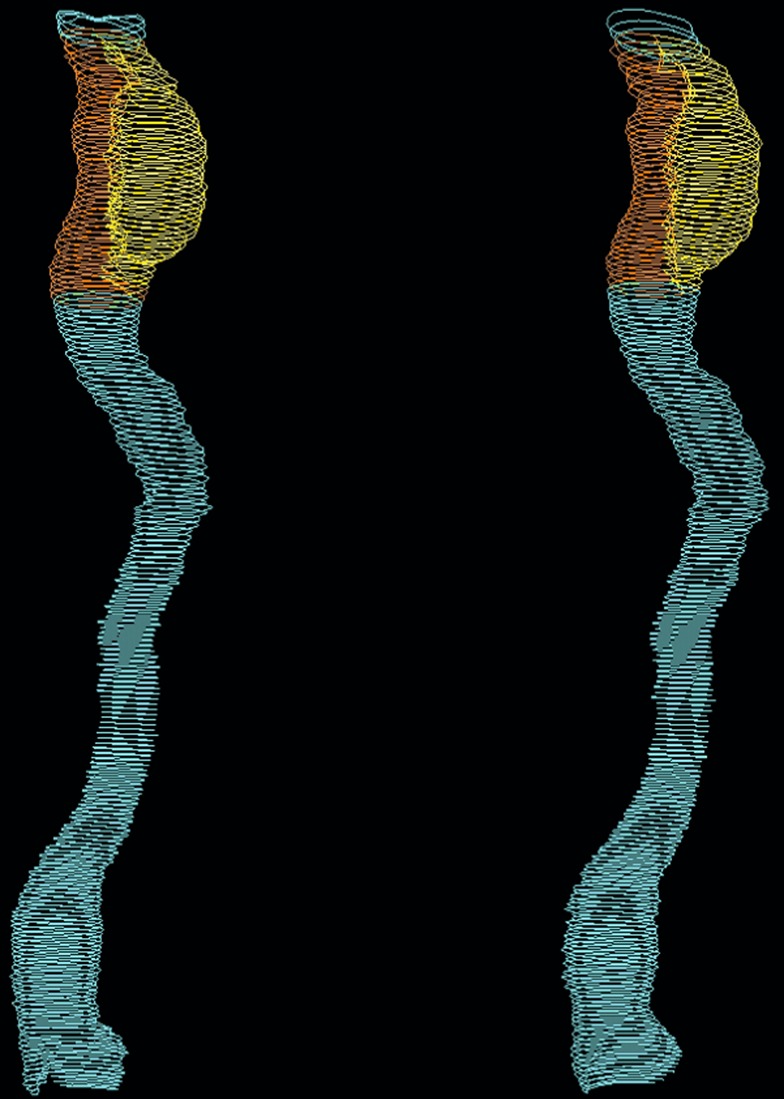
Example of segmentation of operator 1 (left) and operator 2 (right). The separate segmentations are referred to “only vessel” in blue, “aneurysm sac” in yellow, and “vessel adjacent to aneurysm sac” in orange. Total volume of *method 1* (entire ICA) is calculated by adding up the volumes of all three separate segmentations. Total volume of *method 2* (solely  the ECAA) is calculated by adding up the volumes of “aneurysm sac” and “vessel adjacent to aneurysm sac”.

Saccular aneurysms: All saccular ECAAs were measured similarly. First, volume of just the aneurysm sac was measured. In case the outer wall of the aneurysm sac adjoined the outer wall of the ICA, volume of the aneurysm sac was additionally measured in combination with the part of the ICA adjacent to the aneurysm neck and sac (Fig. 2B). Hence, both separate segmentations were added to calculate volume of method 2 (Fig. 3).

For a schematic overview of the twenty cases, we refer to Appendix A. The final segmentations as performed with MeVisLab (see next subsection) are included in Appendix B. A case example with color coding and names of separate volumes is shown in Fig. 3.

### Software

In-house developed software (by H.K.) implemented in MeVisLab (MeVis Medical Solutions AG, Bremen, Germany) was used to manually trace the ICA and borders of the ECAA. This program provided a user-friendly spline drawing and editing technique, with which we were able to follow the borders of the structures closely. All volumes were manually segmented from outer to outer wall, as is standard in segmentation of abdominal aortic aneurysms^4–6^. All cases were segmented on axial view (sagittal and coronal views were only used to identify structure borders). Volumes (in mL) were obtained by multiplying the size of the segmented volume by the voxel size of the acquired image.

### Study design

Two operators (E.V., C.L.) independently segmented each of the 40 scans according to the measurement methods 1 and 2 in the predefined protocol. Prior to measurement of the 40 scans, both operators measured three test scans in order to familiarize with the program. The results of these test segmentations were compared to overcome potential differences in protocol interpretation. Afterwards, the twenty cases were offered to the operators in randomized fashion in order to prevent a potential effect from learning. Of each case, the first and second scans were measured in succession. A random sample of five ECAAs was selected for both operators to be measured twice according to method 2.

### Imaging

A 64-slice or 128-slice CT scanner (Philips Brilliance; Philips medical systems, Best, the Netherlands) was used to acquire the CTA scans. The carotid arteries were visualized from the aortic arch up until the skull base. The median slice thickness was 0.9 mm (range 0.6–3.0 mm), increment 0.33 mm, pitch 0.609 mm, and collimation 64 × 0.625 mm. Tube voltage was 100–120 kV and tube current 17–300 mA. Fields of view were set per patient. Injection of 65 milliliter intravascular contrast (ultravist 300, Schering, Berlin, Germany) was followed by a saline bolus of 40 milliliter, both at a flow rate of 6 milliliters per second.

### Outcomes and statistical analysis

Crude volumes were calculated for method 1 (entire internal carotid artery ipsilateral to the ECAA) and method 2 (solely the ECAA).

The primary outcomes were measurement reliability and agreement of the protocol. Interoperator reliability of each volume was calculated by the intraclass correlation coefficient (ICC; model: two-way mixed, type: consistency). This was done for the entire ICA (method 1), as well as for the ECAA (method 2, A and B). An ICC of 1.0 equals perfect reliability, an ICC of 0.81–0.99 excellent reliability, and an ICC of 0.61–0.80 substantial reliability^10^. Intraoperator reliability for measurement method 2 was calculated for both operators for the random sample of five scans. Inter- and intraoperator agreement was assessed by use of Bland-Altman analysis.

In addition, Dice Similarity Coefficients (DSCs) and modified Hausdorff Distances (HDs) were calculated for measurement methods 1 and 2, to assess similarity of the segmentations. The DSC is a measure of spatial overlap of two segmentations, with perfectly overlapping segmentations resulting in a Dice score of 1, and a complete lack of overlap resulting in a Dice score of 0^11^. The HD measures the boundary distance in mm between segmentation contours, with a HD of 0 mm meaning that the two segmentations are identical^12^. We used a modified HD, using the 95^th^ percentile of the distances over the full segmentation to be less sensitive to outliers^13^. The amount of disagreement that was considered acceptable between segmentations was judged per case, and was mainly dependent on the size of the ECAA and the spatial resolution of the images.

All data analyses were conducted using SPSS version 25 (IBM Corp. Released 2017. IBM SPSS Statistics for Windows, Version 25.0. Armonk, NY: IBM Corp.).

## Results

The median age of the subjects was 51 years (range 36–80 years) at the time of the first CTA scan. 50% of subjects was male, and aneurysms were fusiform in 35% of cases. The median time between the first and second scan was 56 months (range 3–184 months). The median aneurysm diameter reported in the patient records at the time of the first scan was 13 mm (range 9–26 mm) for fusiform aneurysms, and 17 mm (range 7–44 mm) for saccular aneurysms. The time needed to perform the manual segmentations of 40 scans was 32.3 hours, or 49 minutes per scan (as an average of both observers).

### Evaluation of the protocol

#### Interoperator reliability and segmentation similarity

Interoperator reliability scores are summarized in Table 1 and indicate excellent consistency for measurement methods 1 and 2, with all ICCs higher than 0.94. Similarity as measured with the DSC was above 0.88 for method 1 and 2, except for the segmentation “vessel adjacent to aneurysm sac” with a DSC of 0.79. The HD ranged from median 0.801 (interquartile range [IQR] 0.430-7.006) mm for method 1 to median 1.229 (IQR 0.584-2.863) mm for segmentation of the “aneurysm sac”.

**Table 1:** Reliability and similarity of both operators for measurement of the entire ICA (method 1), solely the ECAA (method 2), and the three separate segmentations “only vessel”, “aneurysm sac”, and “vessel adjacent to aneurysm sac” (also see Fig. 3).

	ICC (95% CI)	DSC (median, IQR)	HD, mm (median, IQR)
Method 1	0.995 (0.991–0.998)	0.913 (0.895–0.954)	0.801 (0.588–1.728)
Method 2	0.996 (0.993–0.998)	0.906 (0.860–0.957)	1.229 (0.663–2.793)
Separate segmentations*			
- only vessel	0.960 (0.926–0.979)	0.885 (0.857–0.899)	0.947 (0.631–1.435)
- aneurysm sac	0.996 (0.993–0.998)	0.903 (0.854–0.955)	1.229 (0.584–2.863)
- vessel adjacent to aneurysm sac	0.941 (0.891–0.968)	0.788 (0.736–0.818)	1.208 (1.033–2.724)

Reliability was expressed as the Intraclass Correlation Coefficient (ICC), similarity as the Dice Similarity Coefficient (DSC) and modified Hausdorff Distance (HD).

Footnotes: *See Fig. 3 for case example and description of the separate segmentations.

Abbreviations: ICC, intraclass correlation coefficient; DSC, Dice similarity coefficient; HD, modified Hausdorff distance; IQR, interquartile range; CI, confidence interval.

#### Intraoperator reliability and segmentation similarity

Intraoperator reliability for measurement method 2 was calculated for both operators for the random sample of five ECAAs, and was close to perfect with an average intraoperator reliability of 0.999. Similarity of segmentations as measured with the DSC was above 0.93 for both operators, and HD values were below 1 mm for both operators (data not shown).

#### Interoperator agreement

**Figure 4:**
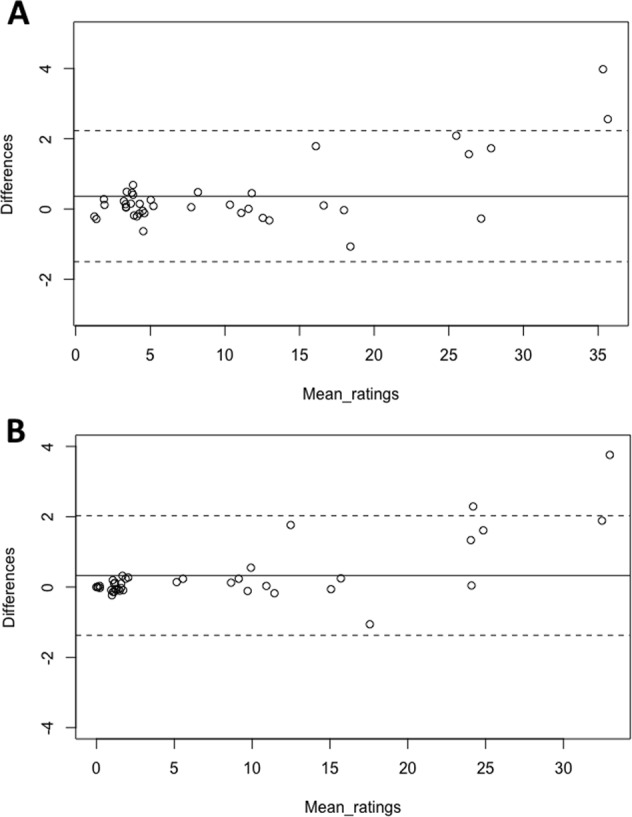
Bland-Altman plots showing agreement of two operators on the volume measurement of 40 scans according to method 1 (**A**) and method 2 (**B**). The line in the middle represents the mean difference of the volume (in mL) between the two operators, and the dashed lines represent the upper and lower limits of agreement (mean difference ±1.96 × standard deviation).

Bland-Altman plots were constructed to assess presence of systematic differences between both operators for method 1 and 2 (Fig. 4A,B, Appendix C). All plots reveal approximately normal differences, as 95% of all differences fell within the limits of agreement. However, proportional bias can be observed in all plots except “only vessel”, since the difference in volume increased with the mean volume.

The measurements that fell outside the upper limit of agreement were cases 7a and 7b for measurement method 1, and cases 3b and 7b for measurement method 2 (Fig. 4). These cases contained aneurysm sacs with a significant amount of thrombus, which hampered identification of the aneurysm outer wall.

## Discussion

In this study, we developed a new protocol for volumetric assessment of ECAAs, and demonstrated that this protocol can be applied in a reliable manner with excellent ICC, DSC and HD values.

The use of volume measurements in addition to diameter measurements for detection of aneurysm growth has been a topic of interest in multiple vascular territories. Literature on abdominal aortic aneurysms showed that volumetric measurements may be superior to conventional diameter measurements for detecting size changes as a follow-up tool after endovascular repair^14,15^, as well as untreated aneurysm surveillance^16^. In intracranial aneurysms, a moderate correlation of volume and diameter at best was only found in predominantly saccularly shaped aneurysms, and not at all in fusiform aneurysms^17^. However, despite abundant evidence, volume assessment of aneurysms over diameter measurements to assess size is still not standard practice in most institutions. This is largely because volume assessment is time consuming, requires dedicated software and skilled technicians, and carries significant operator variability^18^. Our volume measurement protocol for ECAA was applied by two operators with excellent levels of reliability and agreement, even though the current manual segmentation process was time-consuming. However, it was designed as a step-up towards development of a (semi)automatic volume measurement tool. This future application aims to minimize the time required to perform the volume assessment, and will bypass the need for skilled technicians. It is crucial to develop a robust and feasible tool to monitor the aneurysm sac, by measuring growth and geometrical alterations, and hereby aid in selection of patients who should undergo intervention to prevent future adverse events.

Our protocol comprised two measurement methods, segmentation of the entire ICA and solely the ECAA. The specific segmentation of solely the ECAA was demonstrated to be reproducible with excellent interoperator reliability. Segmentation of the entire ICA had been included in the protocol to overcome expected difficulties in identification of aneurysm starting point and terminus. However, even though in case of fusiform aneurysms the cutoff value of >125% enlargement on axial slices on CTA was pragmatic and might be considered arbitrary, this did not hamper accurate and similar segmentation between operators.

Median HD values were below 1.3 mm for all methods of segmentations, which translated into a difference of 2 mL in volume according to the Bland-Altman plots. The outliers were mostly explained by the two aneurysms which contained a large amount of thrombus alongside the vascular wall. An additional analysis without these two thrombus-containing aneurysms revealed a median HD value of 1.1 mm, and a corresponding difference of 1 mL in volume. This means that the measurement error is 1–2 mL (depending on in- or exclusion of thrombus-containing aneurysms), and that a difference in ECAA volume should be at least 1–2 mL to allow detection with our volume measurement protocol. We consider this difference negligible for all aneurysms irrespective of their initial size, and believe this measurement error is small enough for our protocol to allow adequate detection of ECAA growth.

For this project we had to face several challenges. First, although we use a common definition for ECAA shape, this definition remains somewhat arbitrary, and mixed forms exist next to saccular and fusiform shapes. As our protocol required a different measurement approach for saccular and fusiform aneurysms, it was crucial to establish the ECAA morphology first and start the corresponding measurements thereafter. In addition, even though separate segmentations were reproducible with high ICC, DSC, and HD values (Table 1, Fig. 3), the distinction between ‘aneurysm sac’ and ‘vessel adjacent to aneurysm sac’ might be judged clinically irrelevant. Therefore, both volumes were added up to calculate aneurysm volume (method 2). Second, although significant thrombus formation within an ECAA is relatively rare, some ECAA do contain thrombus which is likely to challenge segmentation of the aneurysm sac (as can be seen in cases 3 and 7 with relatively low interoperator agreements). Therefore, in this stage, our protocol is only workable for ECAA which contain no or minimal thrombus, and future studies need to focus on better visualization techniques to enable volume measurements of the challenging ECAA with significant thrombus. Third, a thick slice 3.0 mm CTA scan is likely to be less accurate than a thin slice 0.6 mm CTA scan for volume measurements, and a discrepancy in slice thickness in two paired scans performed at different time points might introduce bias. Therefore, all CTA scans should have similar thin slice thickness in order to enable accurate conclusions about ECAA growth. Lastly, it is unknown how little changes in head posture during scanning might influence ECAA and ICA volumes. We hypothesize that 3D volume measurements are less sensitive to these postural changes than conventional 2D diameter measurements. Nevertheless, studies are advised to scan patients with their head in locked position to rule out confounding due to different head and neck postures.

## Conclusion

This proposed protocol to measure ECAA volumes revealed excellent inter- and intraoperator reliability and agreement, as well as excellent similarity of the segmentations performed by two independent operators. Future volumetric assessments are recommended to be included in standardized imaging protocols, which should also focus on head and neck posture, and visualization of thrombus and vessel wall. These could be used as a step-up to (semi)automatic volumetric measurements to monitor patients with ECAA.

## Supplementary information

Supplementary information

## Data Availability

The datasets generated during and/or analysed during the current study are available from the corresponding author on reasonable request. The in-house developed software is available for research partners on reasonable request.
