# Supplementary material for: Volumetric assessment of extracranial carotid artery aneurysms
Source: Sci Rep. 2019 May 30;9:8108. doi: 10.1038/s41598-019-44553-0 (PMC6542789; doi:10.1038/s41598-019-44553-0)
Supplement: Supplementary file 1 — Supplementary information [file 41598_2019_44553_MOESM1_ESM.pdf]

Supplementary information

Manuscript title: Volumetric assessment of extracranial carotid artery aneurysms

Authors: EE de Vries[a], CJHCM van Laarhoven[a], HJ Kuijf, CEVB Hazenberg, JA van Herwaarden, MA Viergever, GJ de Borst

[a] both authors contributed equally

Contents

APPENDIX A: *schematic overview of 20 cases* p. 2

APPENDIX B: *final 3D volume segmentations* p. 11

APPENDIX C: *Bland-Altman plots* p. 32

APPENDIX A

Schematic overview of the 20 cases, and subfield segmentations of each case according to measurement method 2 (the ECAA). Each case consists of 2 scans performed at different time points, adding to a total of 40 scans.

Case	Method 2A: Fusiform	Method 2B: Saccular
1	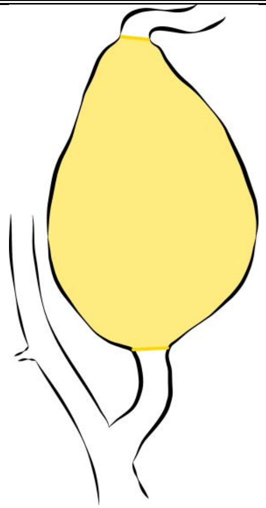	
2		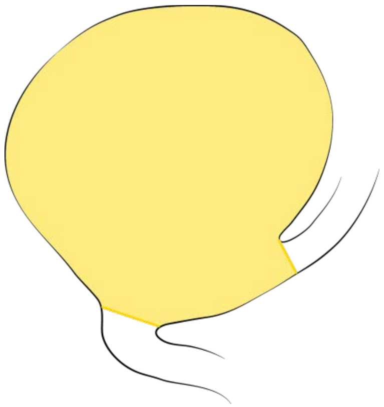

Case	Method 2A: Fusiform	Method 2B: Saccular
3		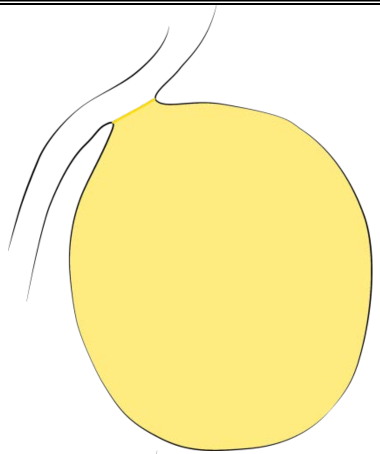
4		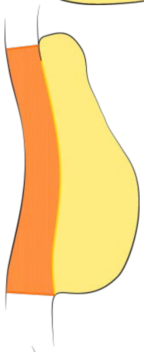
5		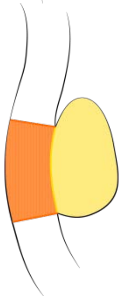

Case	Method 2A: Fusiform	Method 2B: Saccular
6		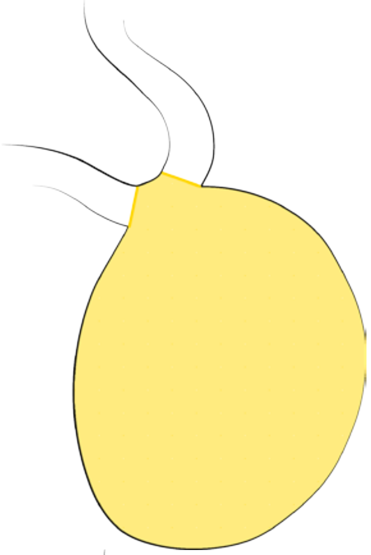
7		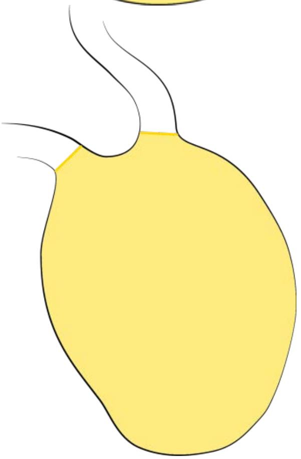

Case	Method 2A: Fusiform	Method 2B: Saccular
**8**		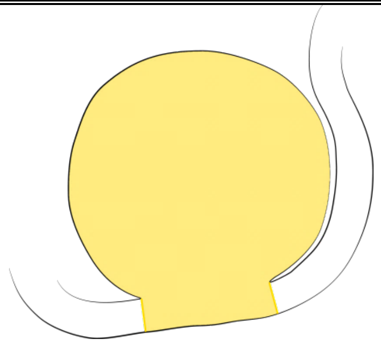
**9**	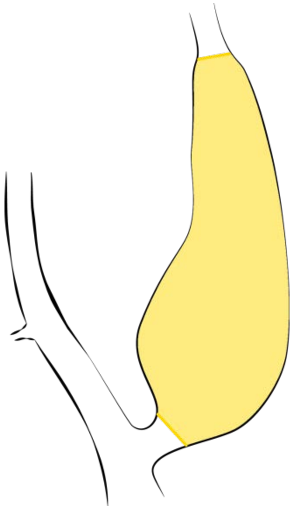	

Case	Method 2A: Fusiform	Method 2B: Saccular
10		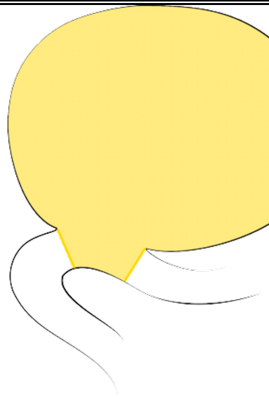
11	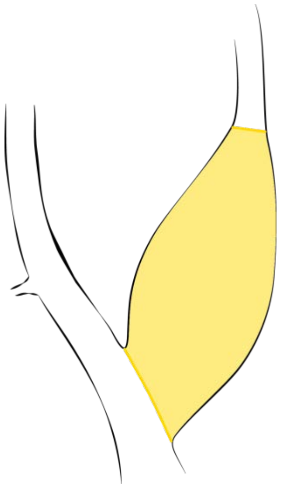	

Case	Method 2A: Fusiform	Method 2B: Saccular
**12**	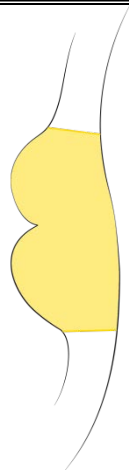	
**13**		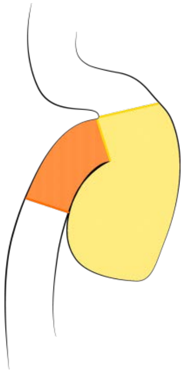
**14**	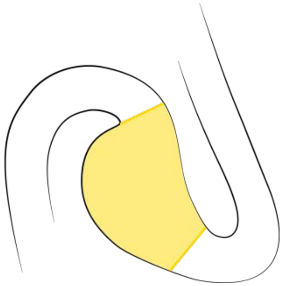	

Case	Method 2A: Fusiform	Method 2B: Saccular
15		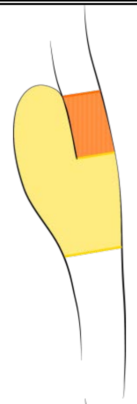
16		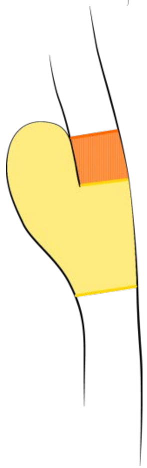

Case	Method 2A: Fusiform	Method 2B: Saccular
**17**	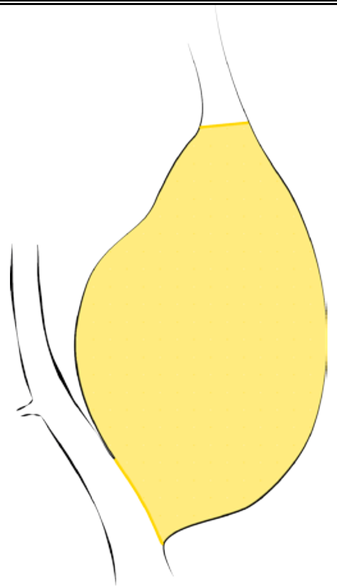	
**18**		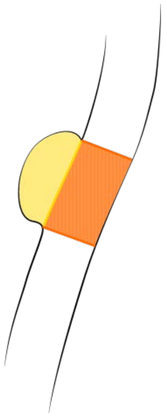

Case	Method 2A: Fusiform	Method 2B: Saccular
**19**		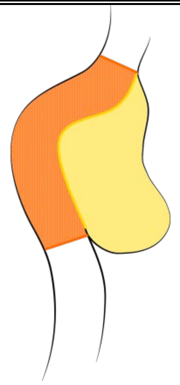
**20**	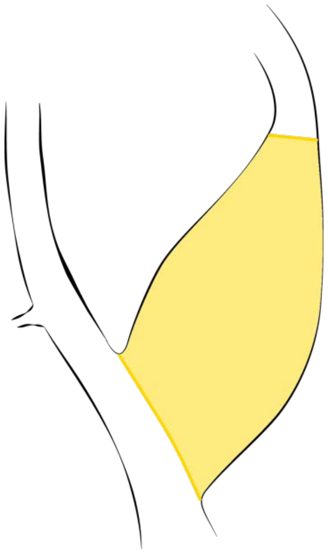	

APPENDIX B

Final 3D volume segmentations of 20 cases. Each case consists of *a* and *b*, indicating the paired scans of the same case performed at different time points. In each scan, the segmentation of observer 1 is shown on the left, the segmentation of observer 2 on the right.

Color coding of subfields:

- only vessel = blue.
- aneurysm sac = yellow.
- vessel adjacent to aneurysm sac = orange.

Case 001a

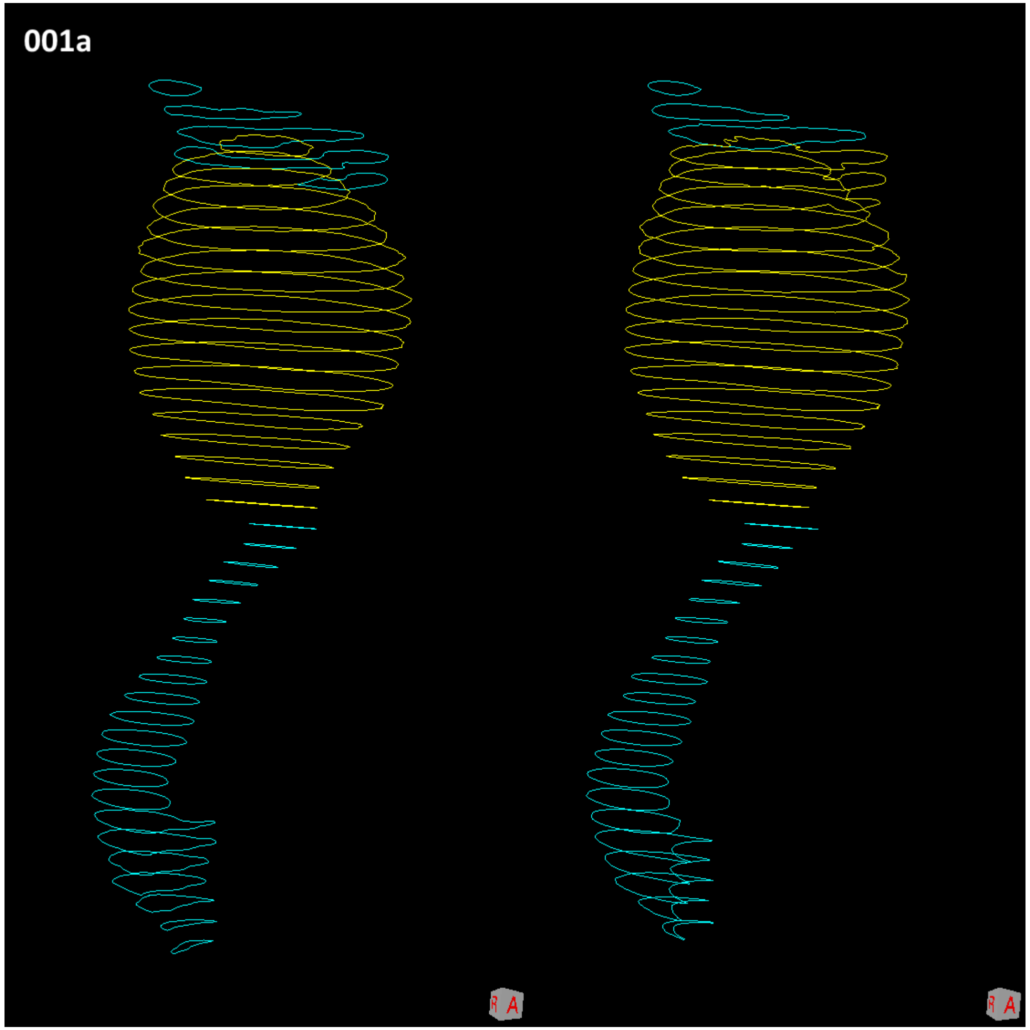

Case 001b

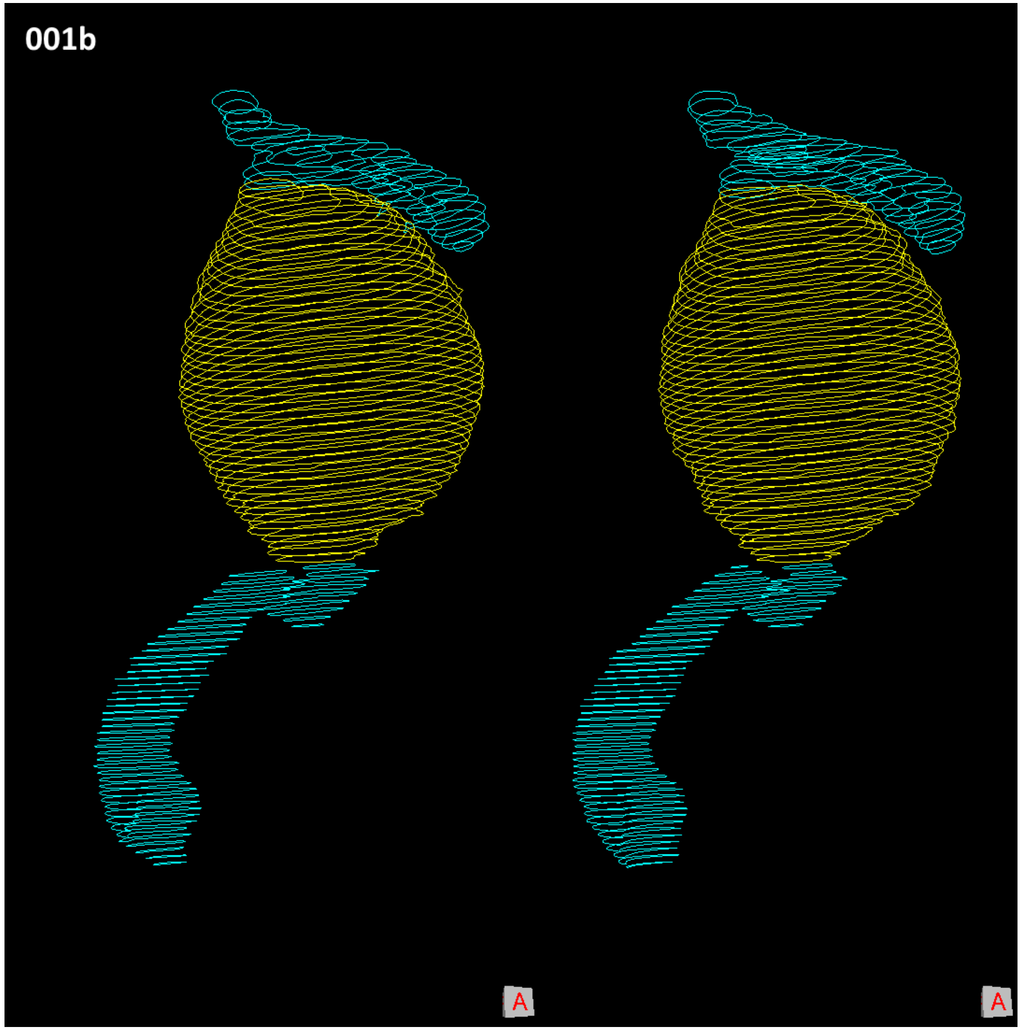

Case 002a

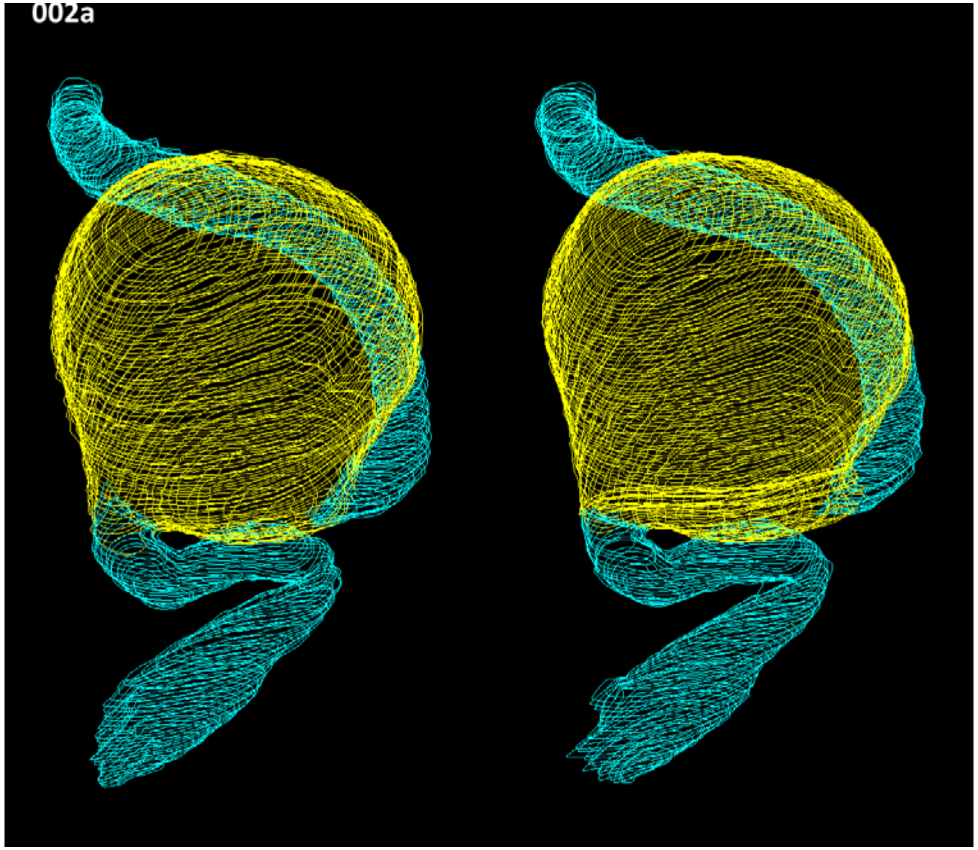

Case 002b

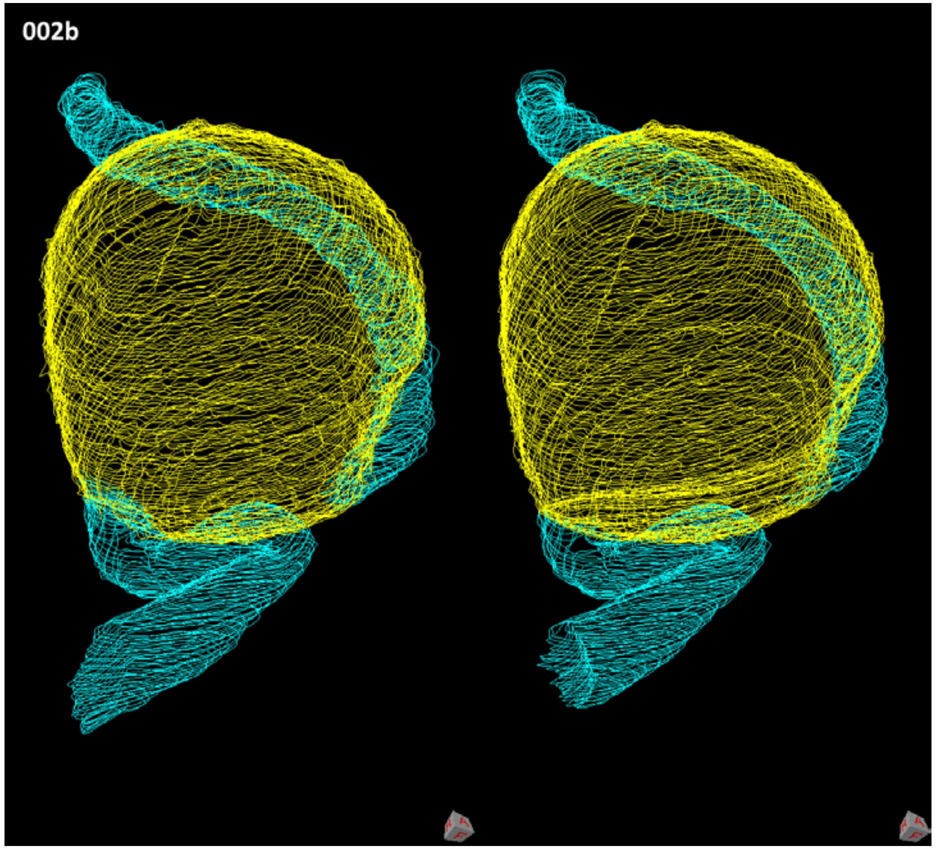

Case 003a

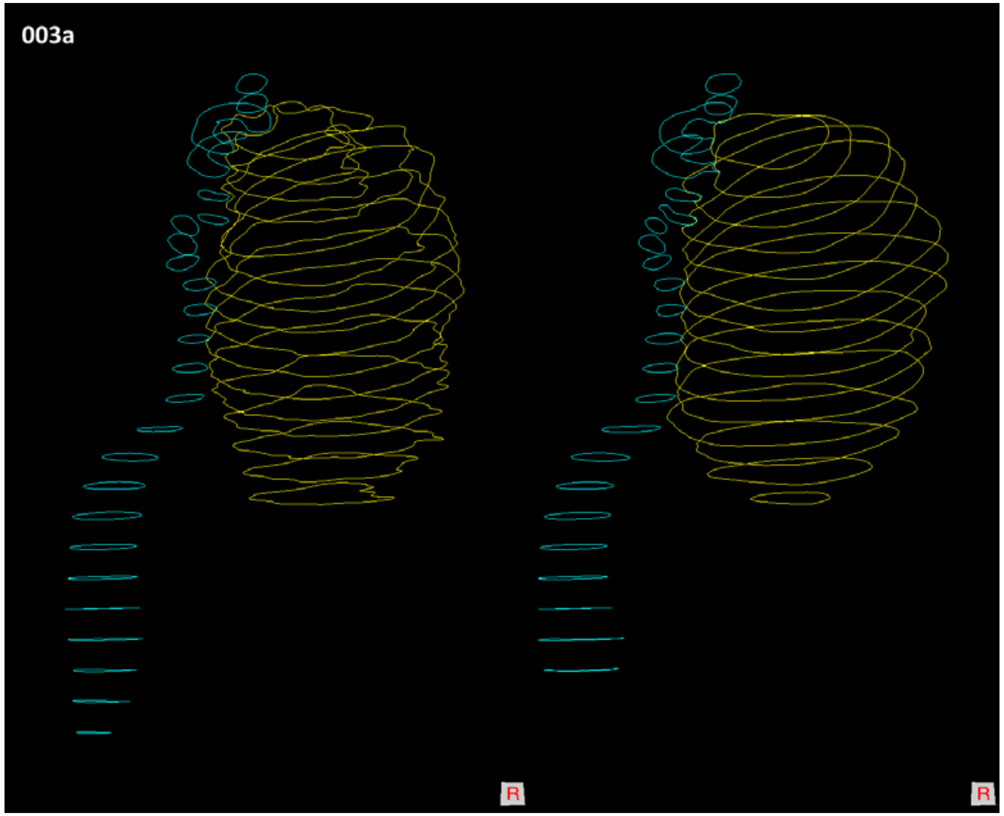

Case 003b

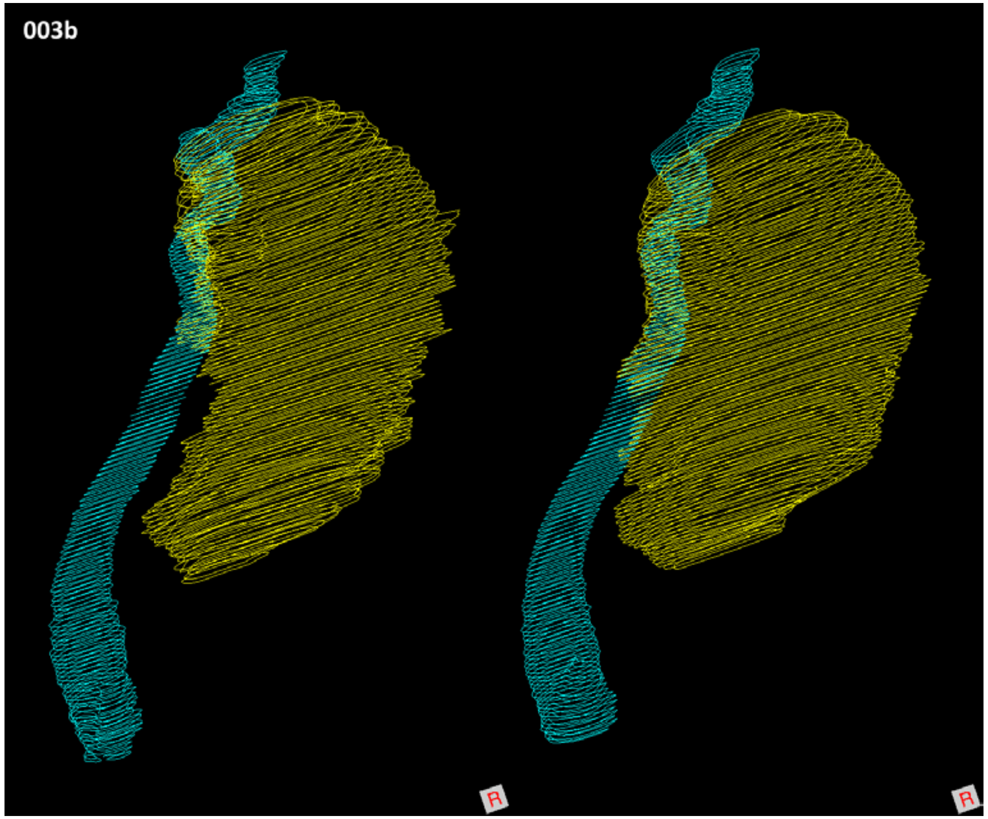

Case 004a

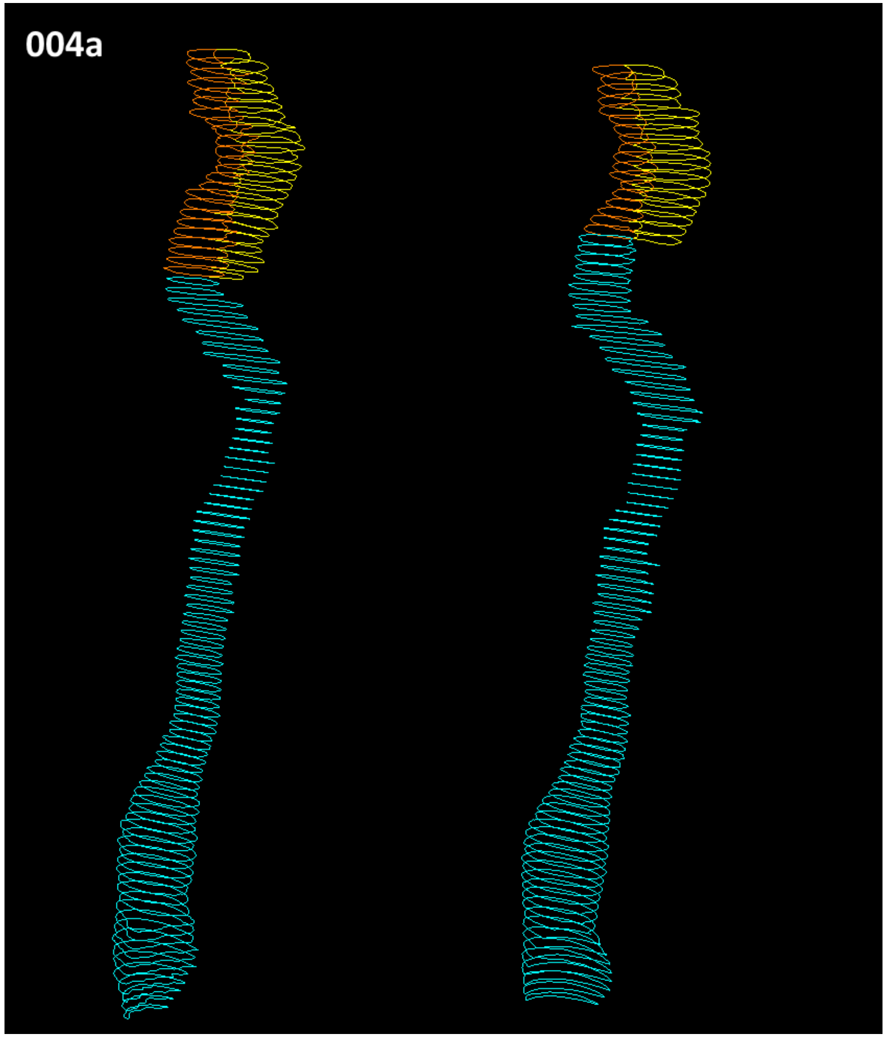

Case 004b

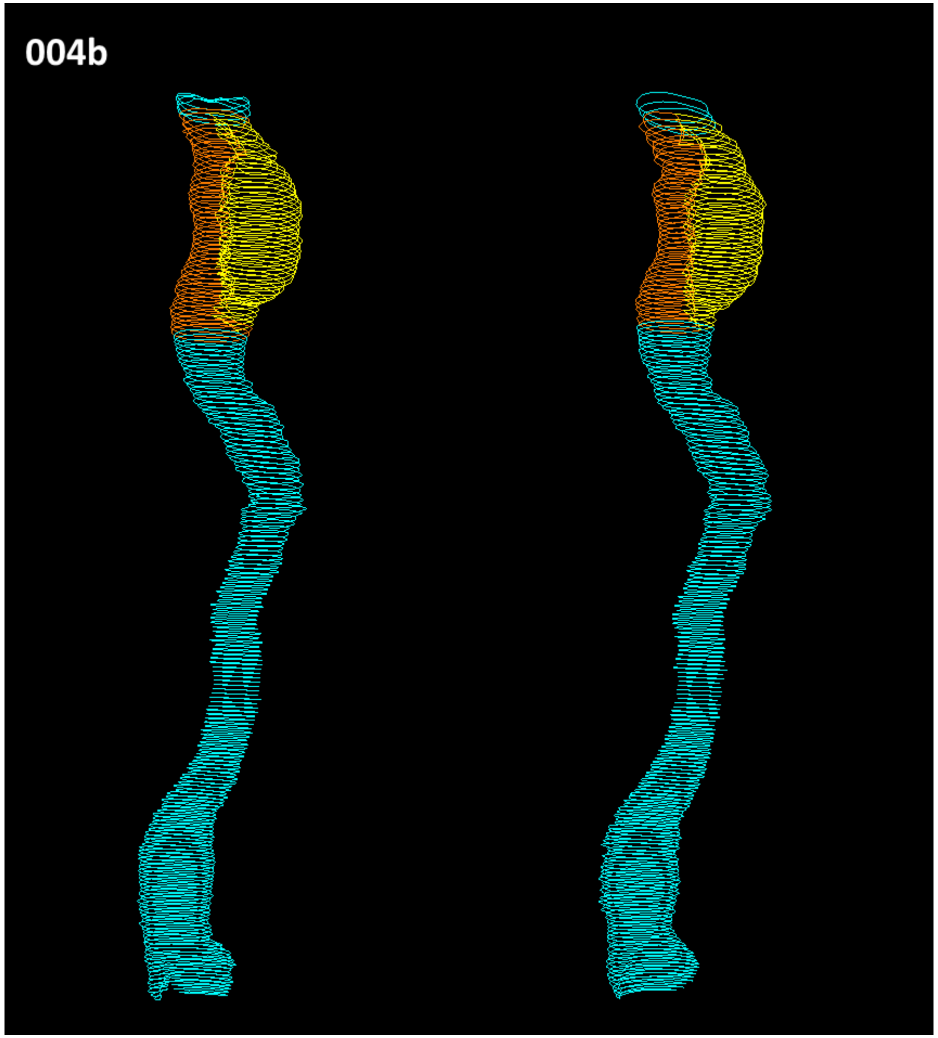

Case 005a

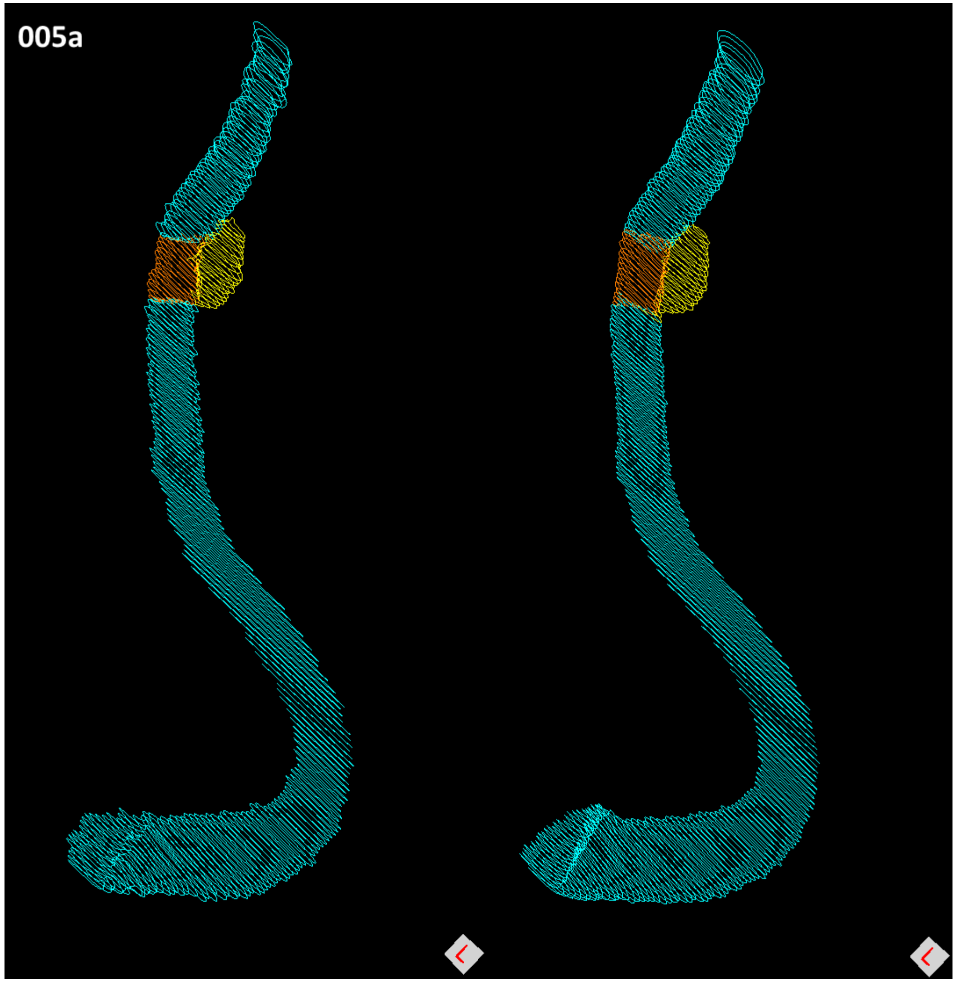

Case 005b

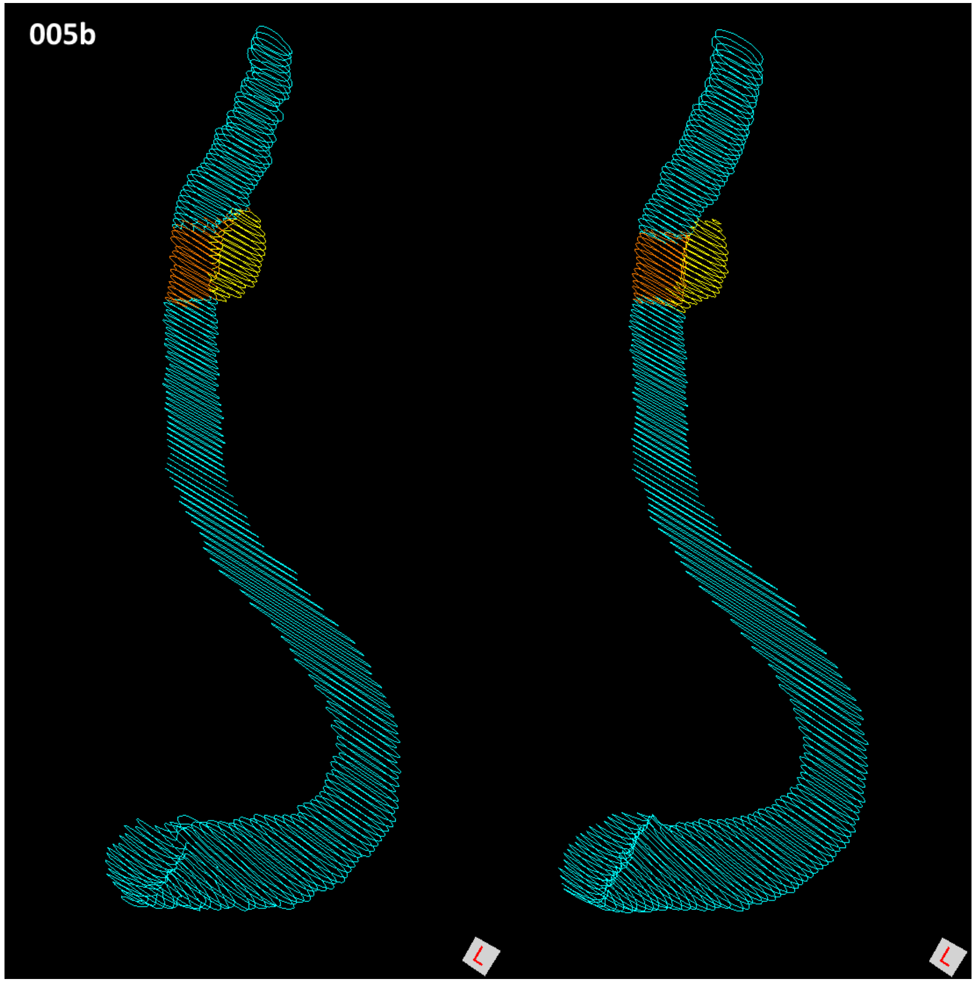

Case 006a

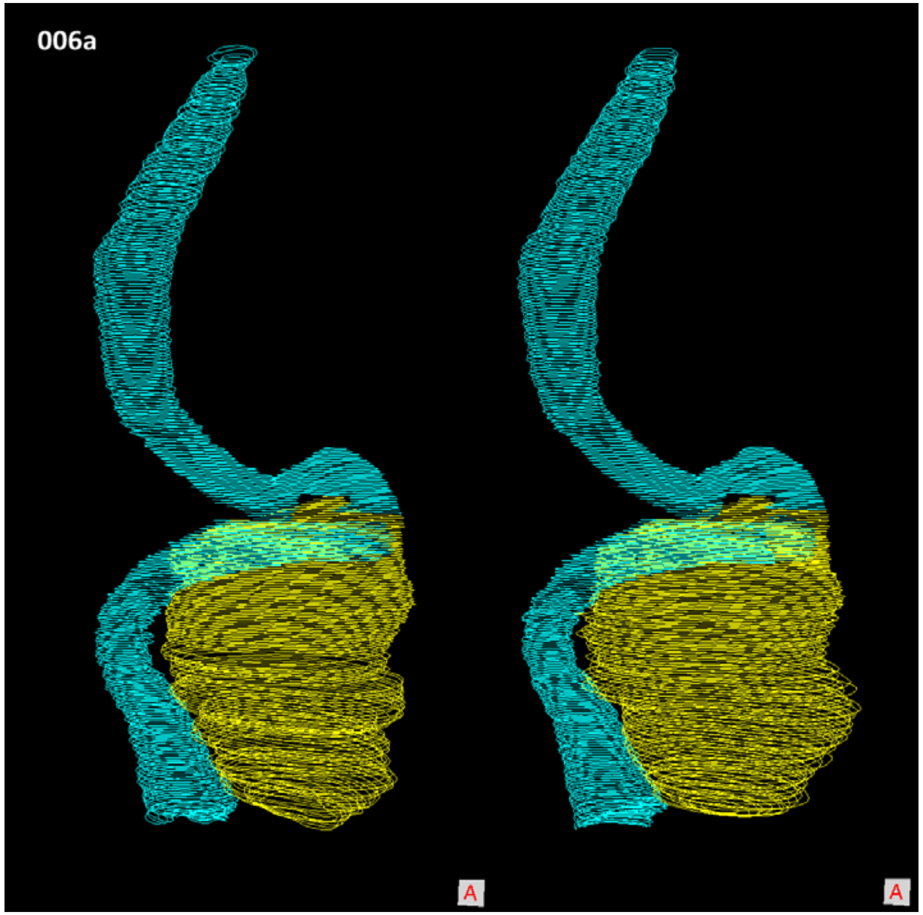

Case 006b

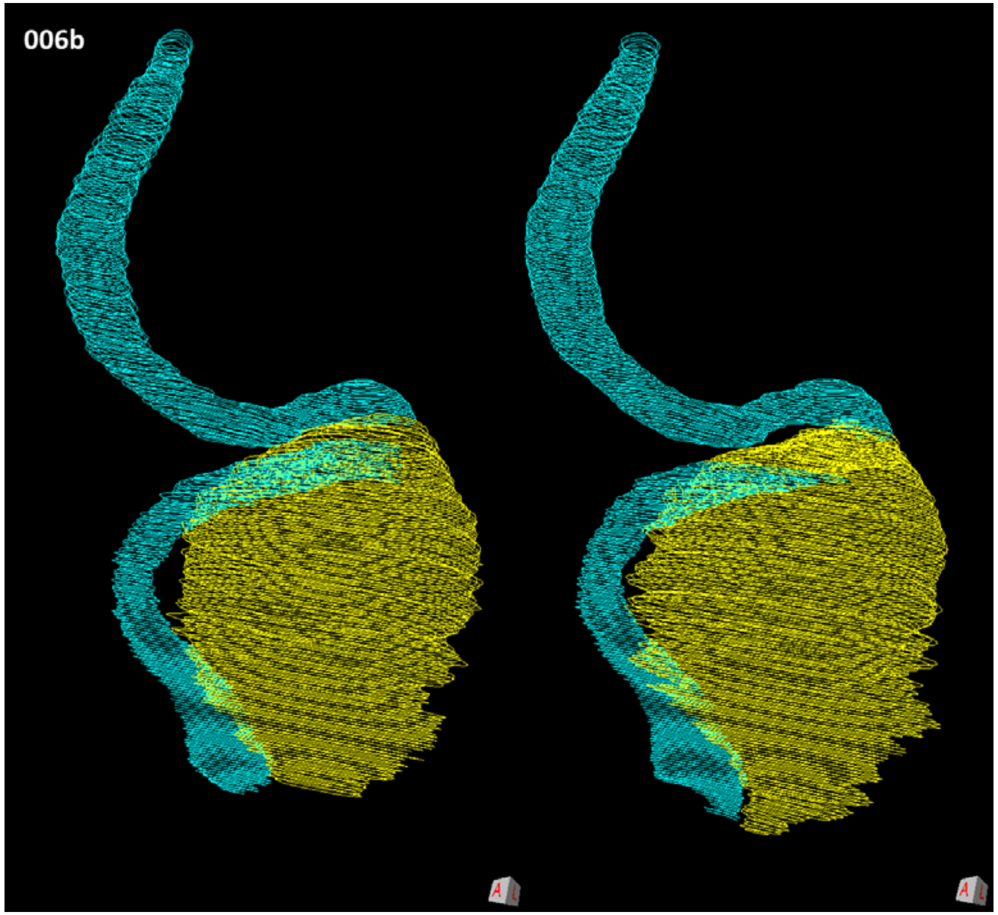

Case 007a

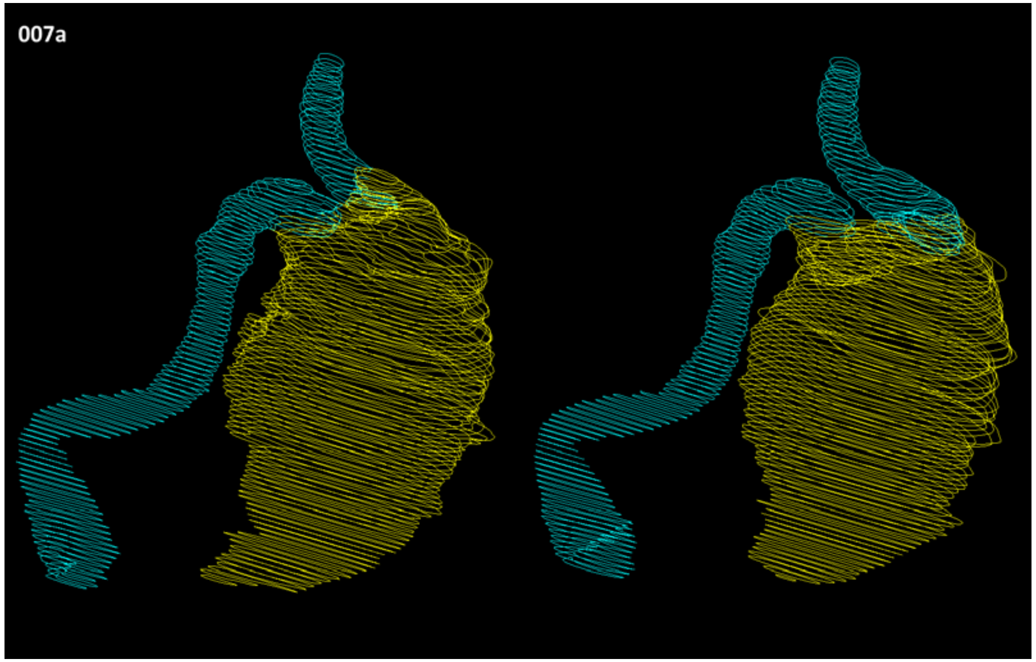

Case 007b

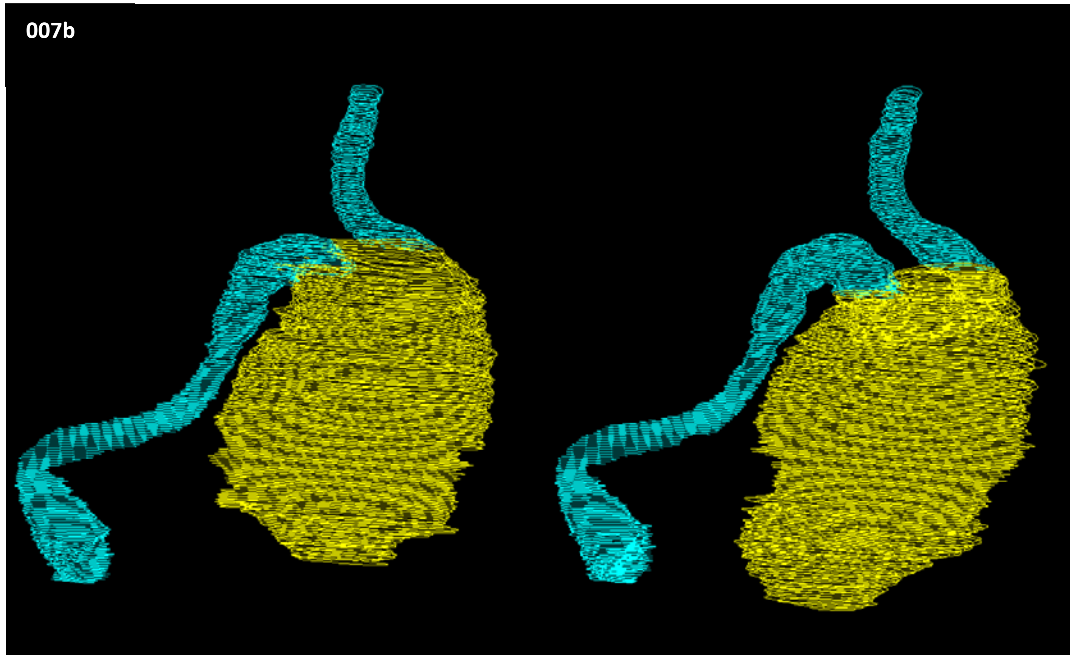

Case 008a

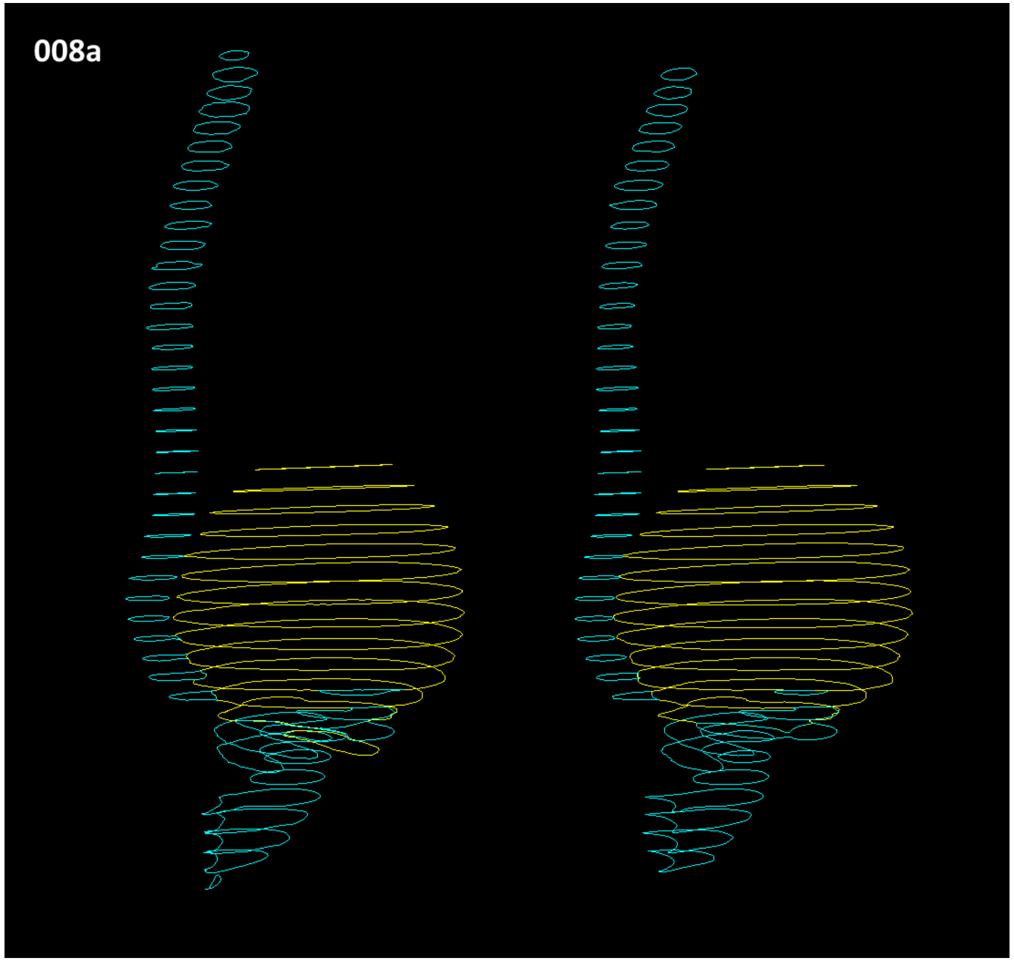

Case 008b

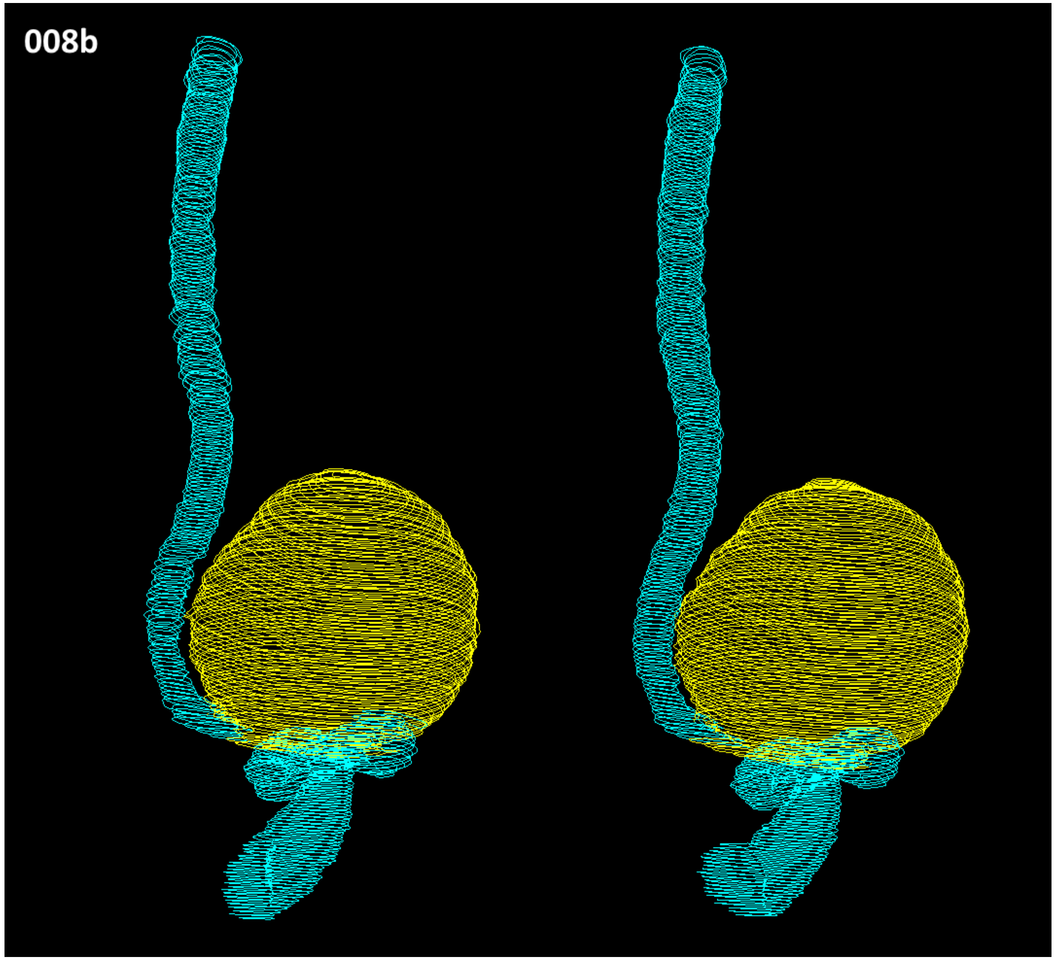

Case 009a

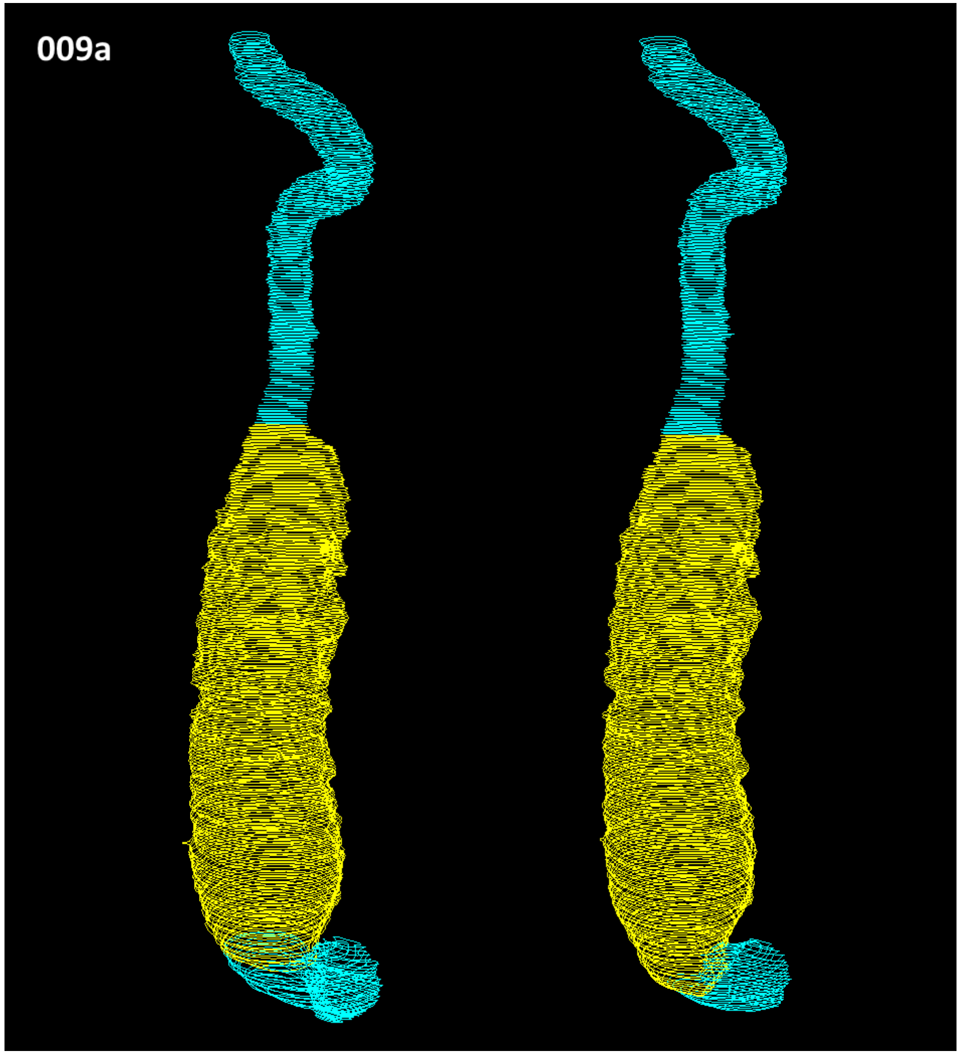

Case 009b

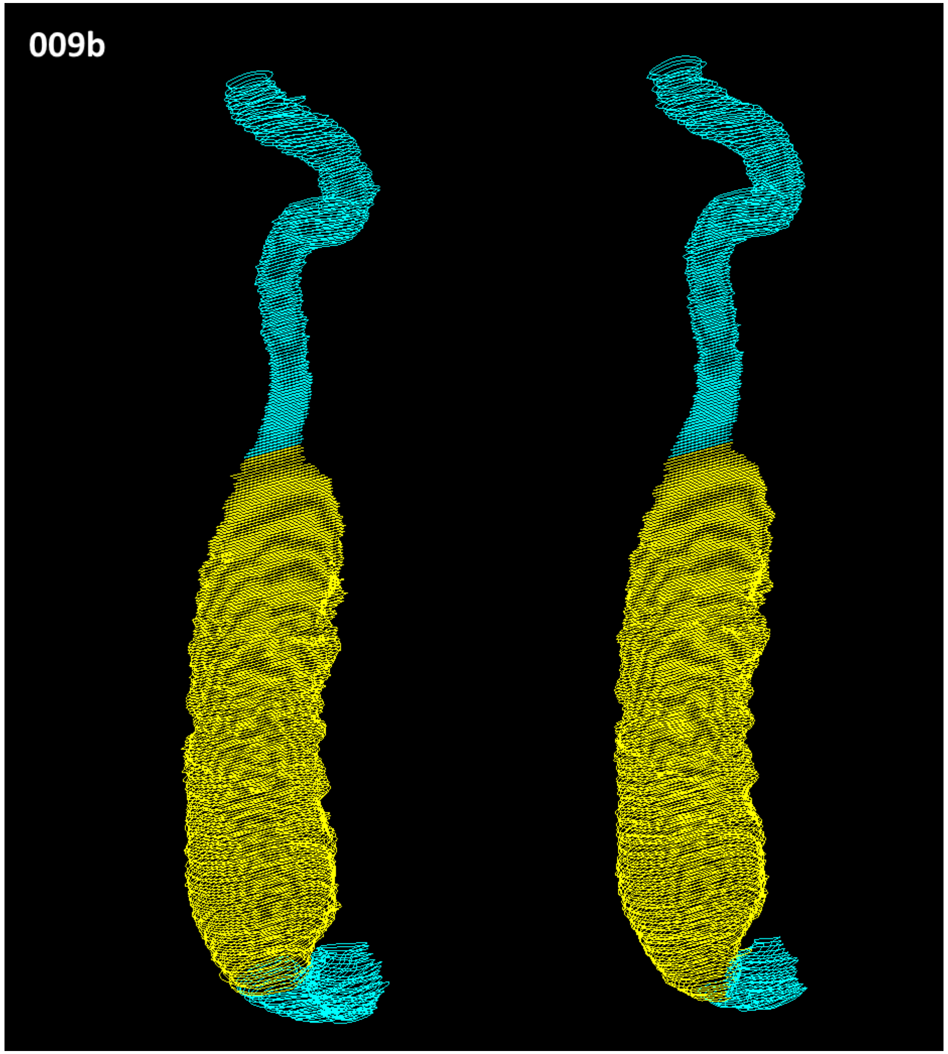

Case 010a

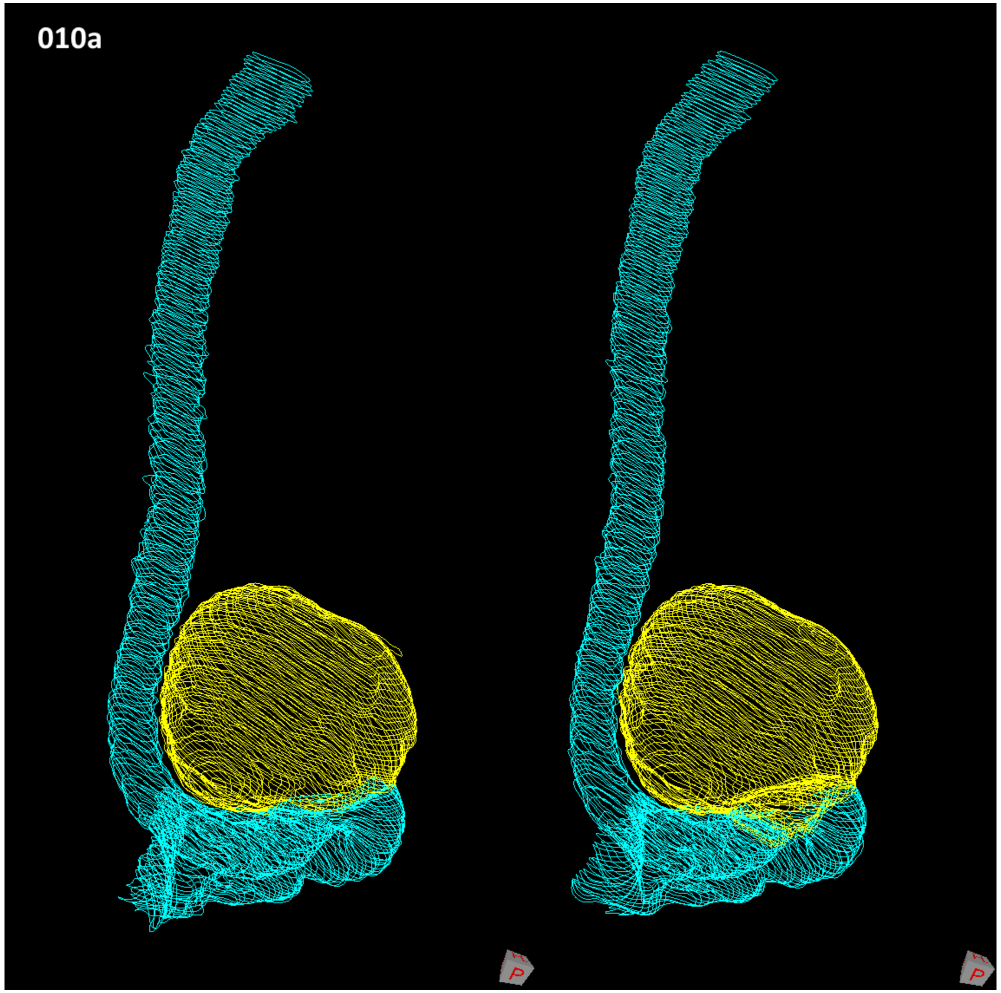

Case 010b

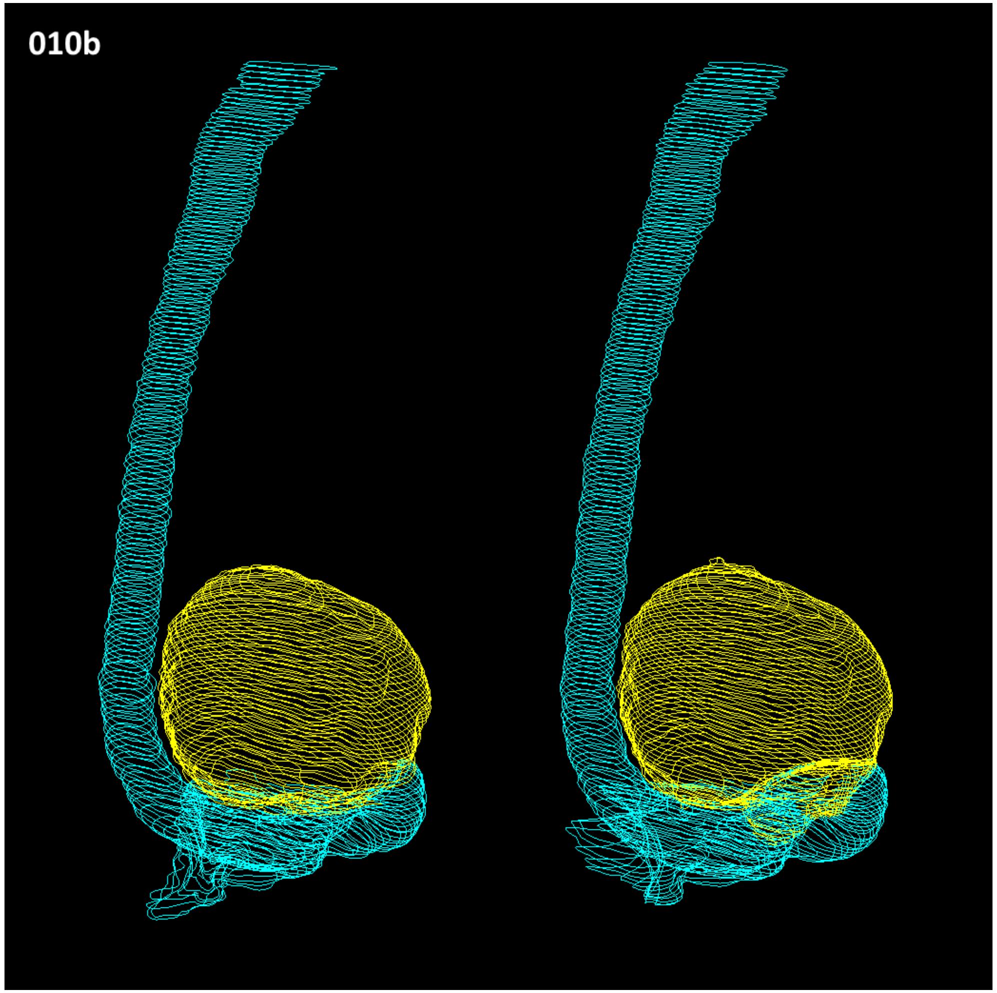

Case 011a

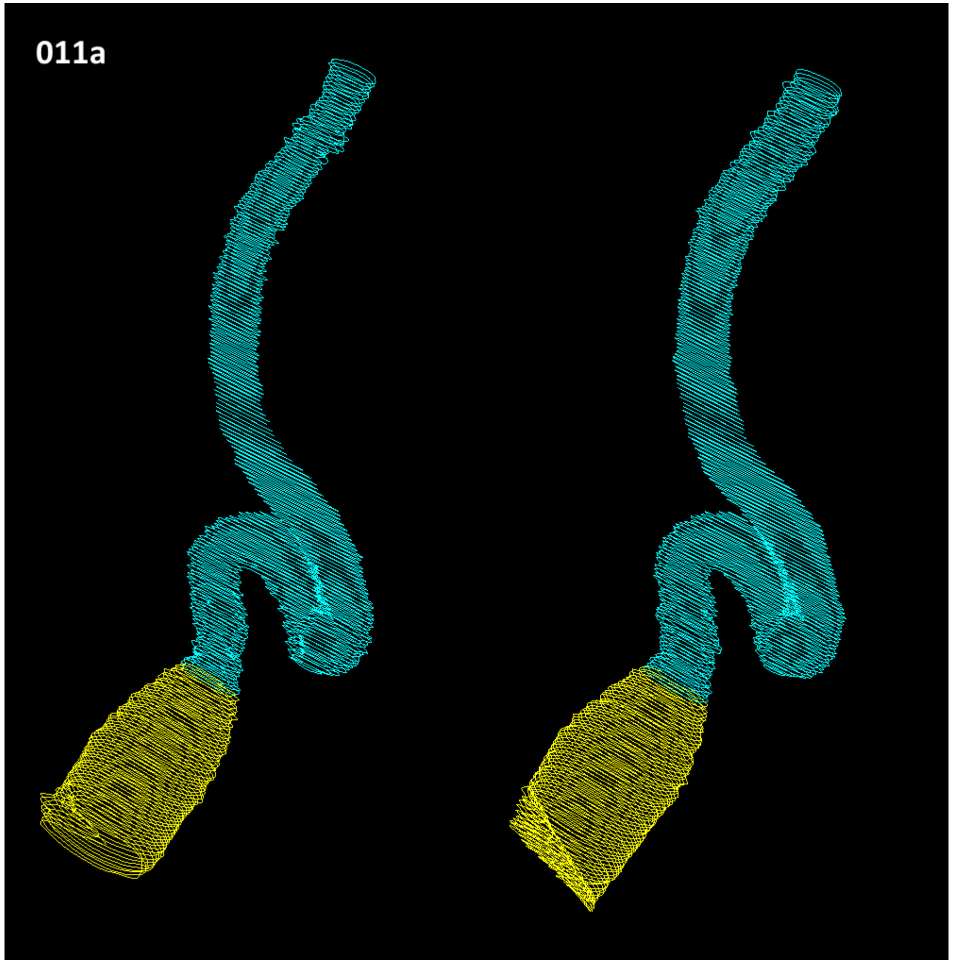

Case 011b

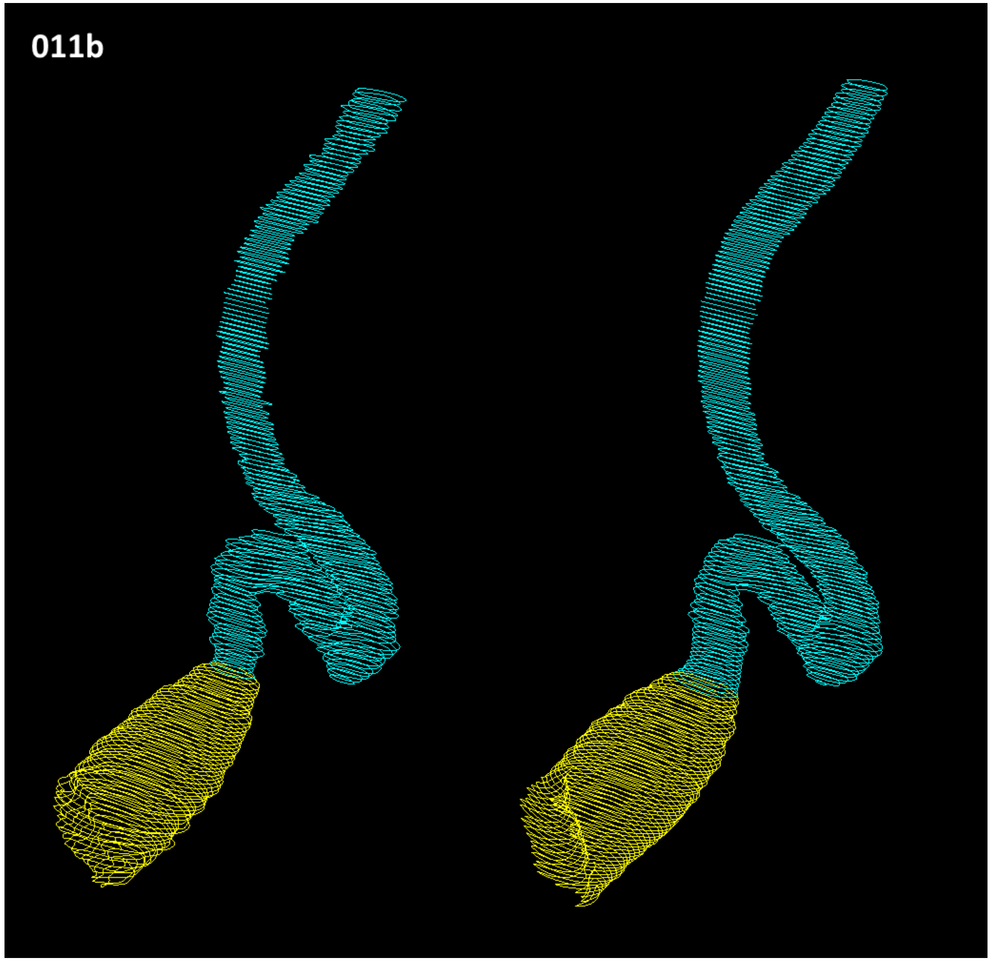

Case 012a

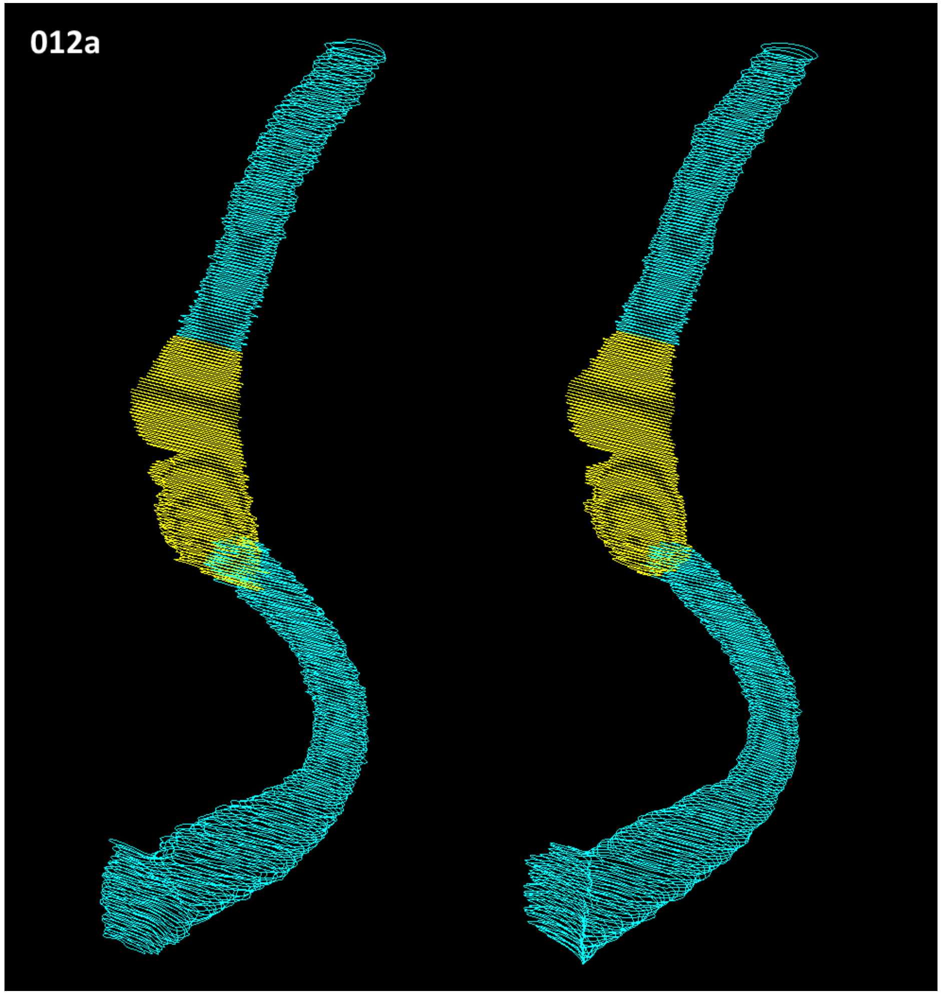

Case 012b

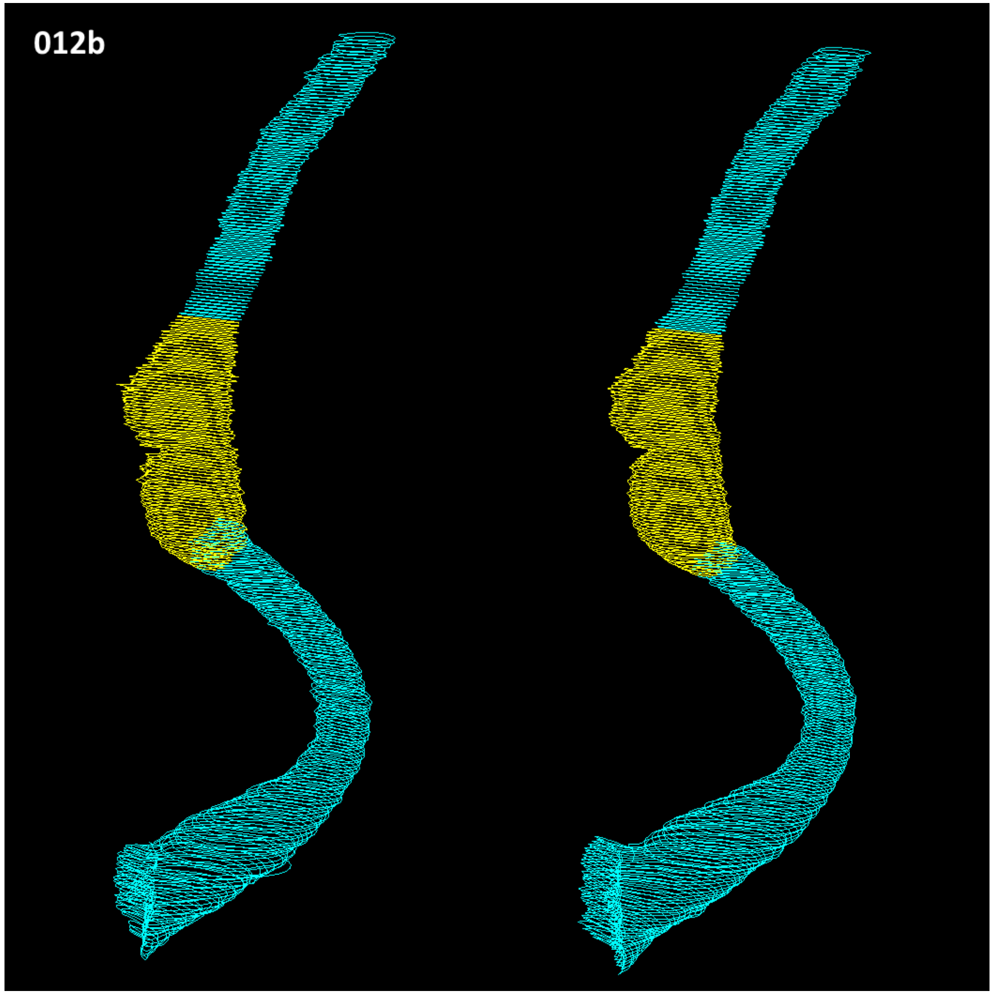

Case 013a

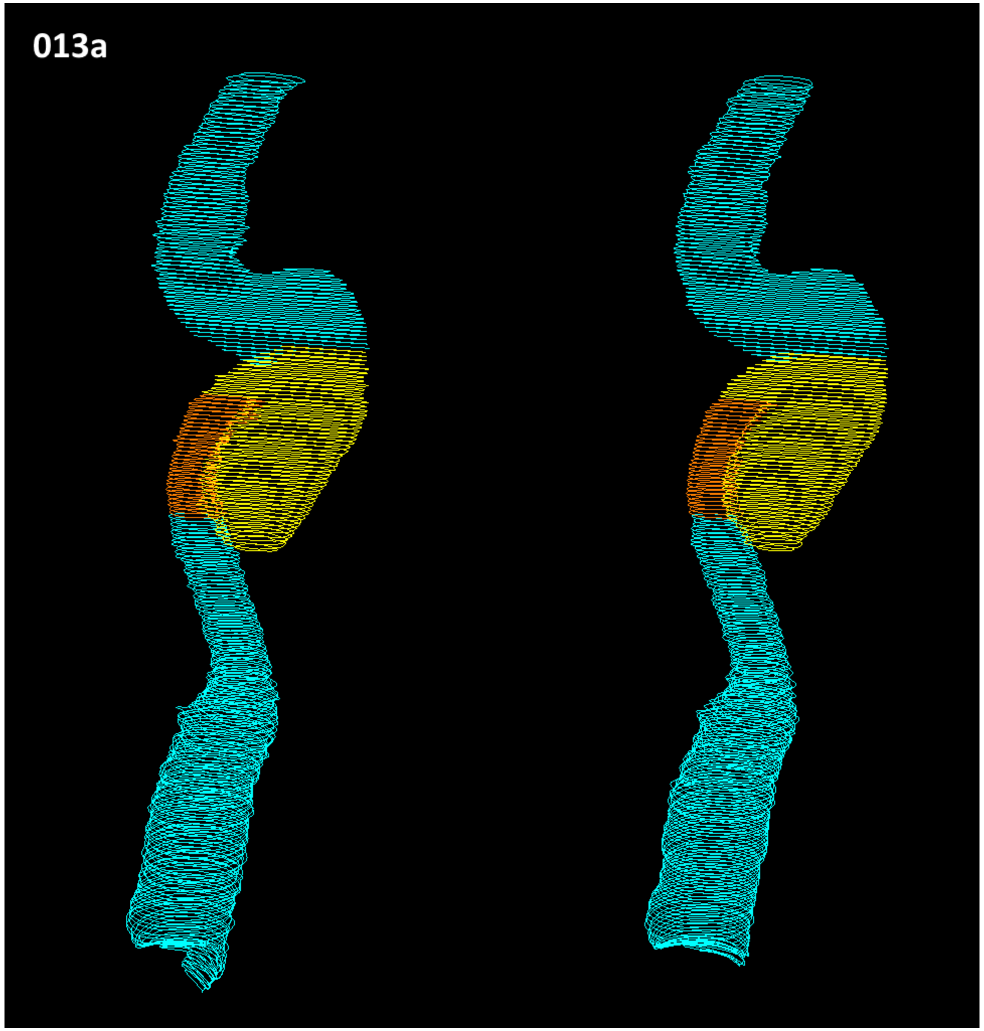

Case 013b

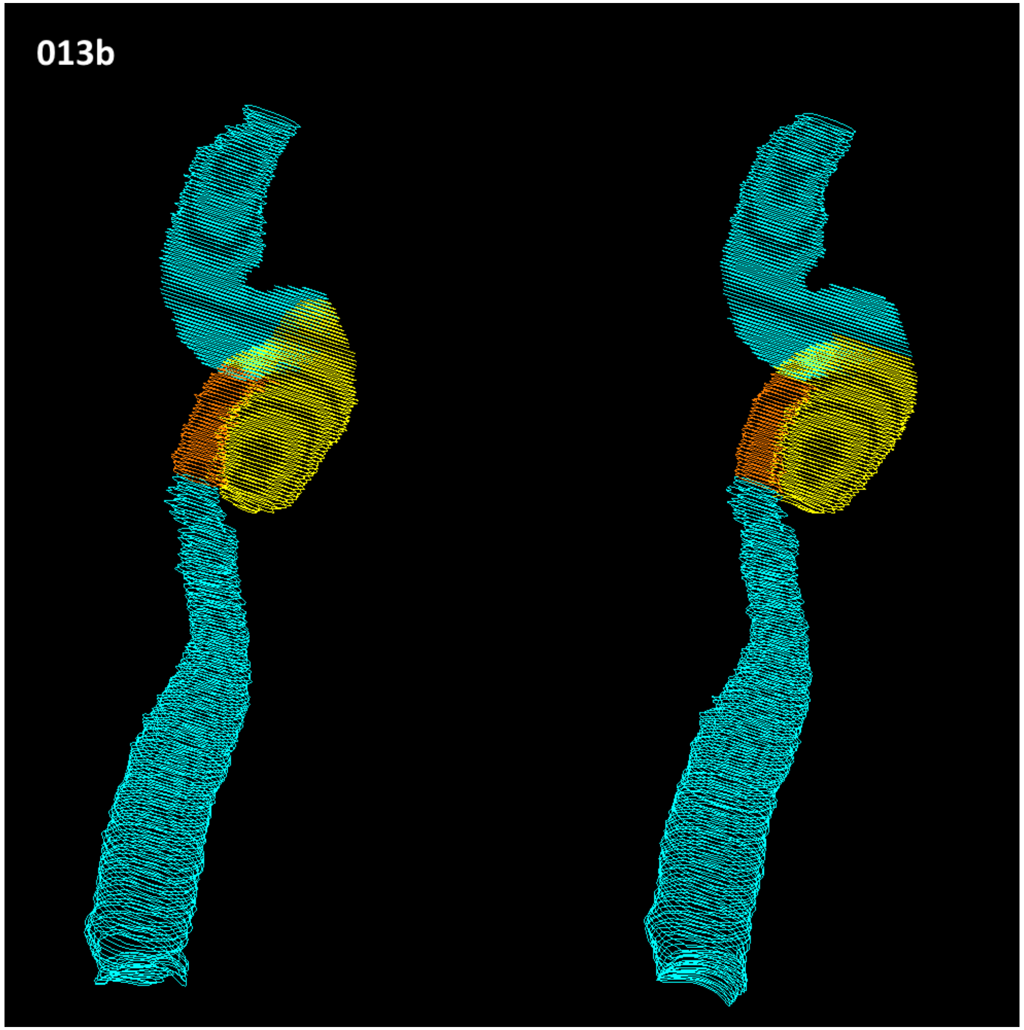

Case 014a

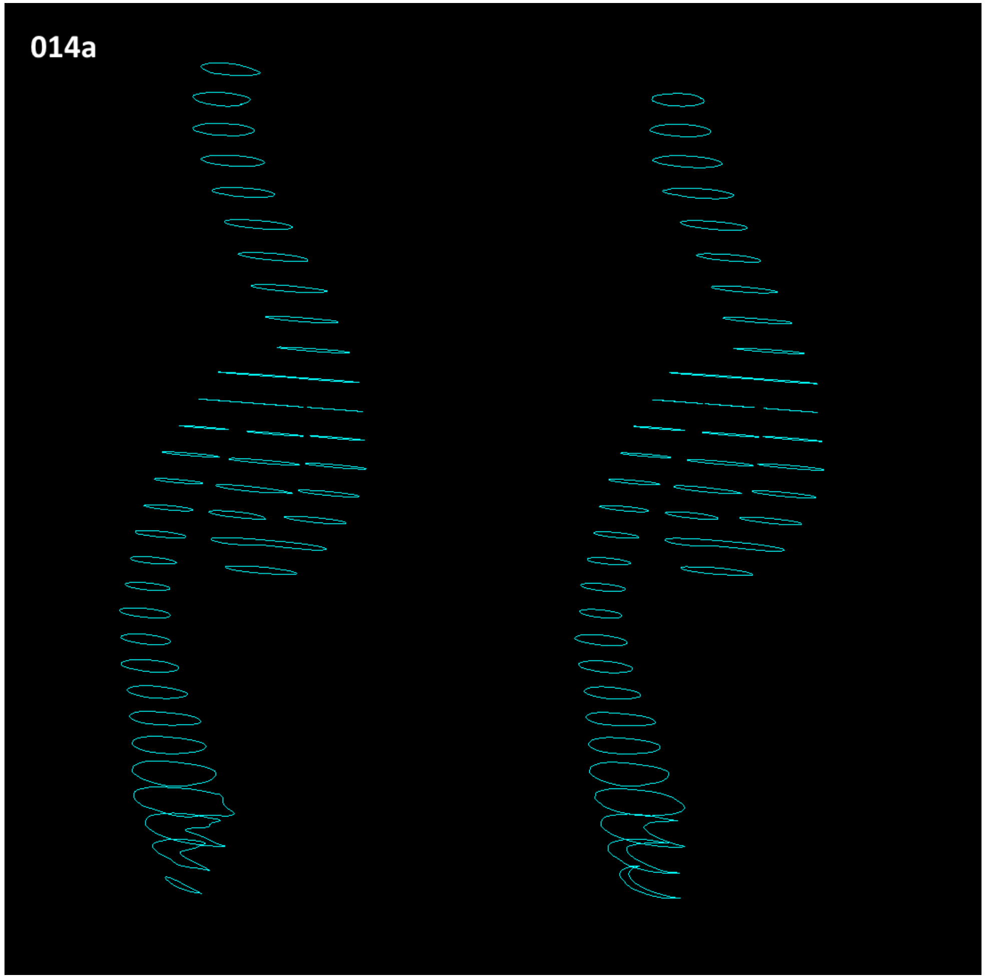

Case 014b

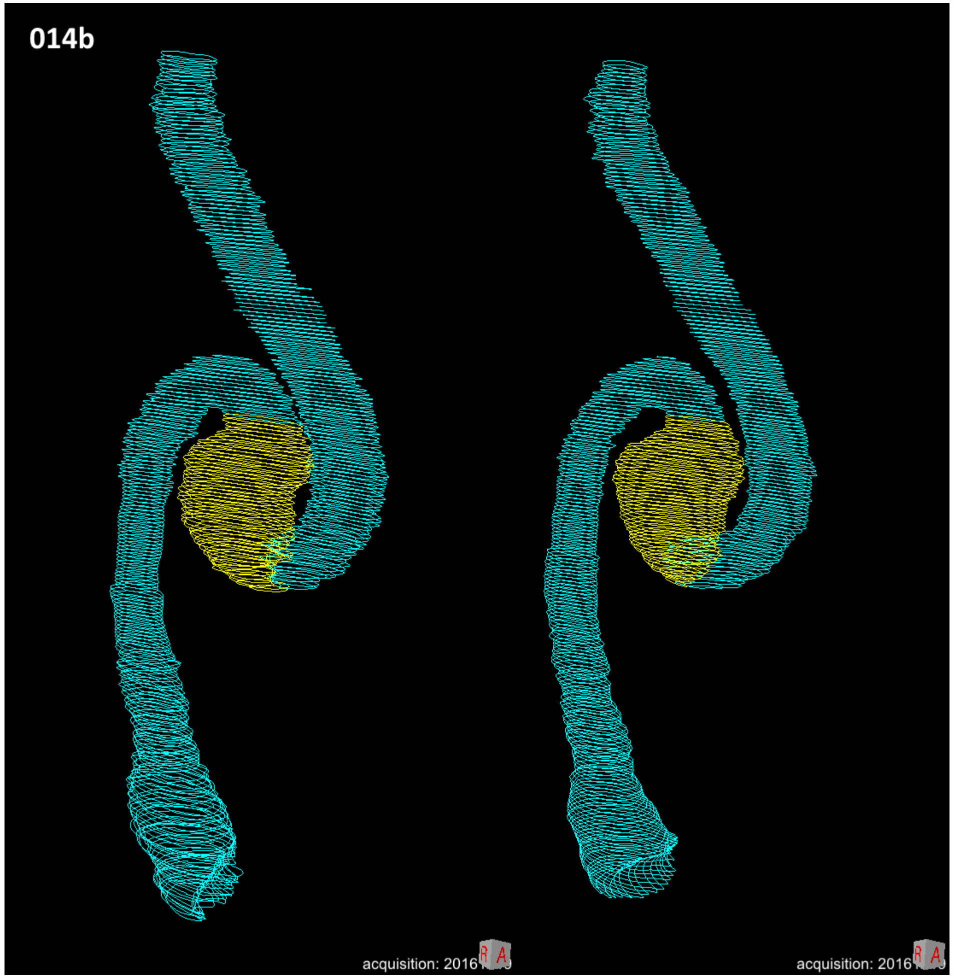

Case 015a

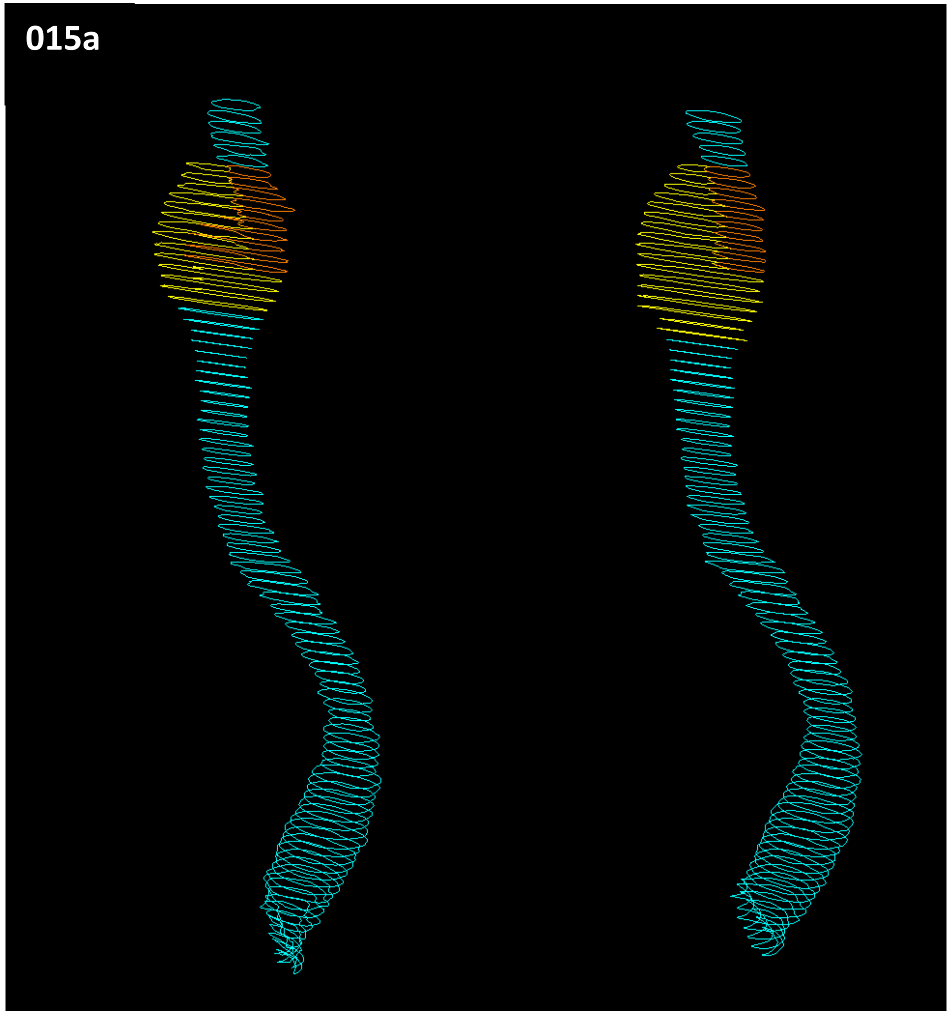

Case 015b

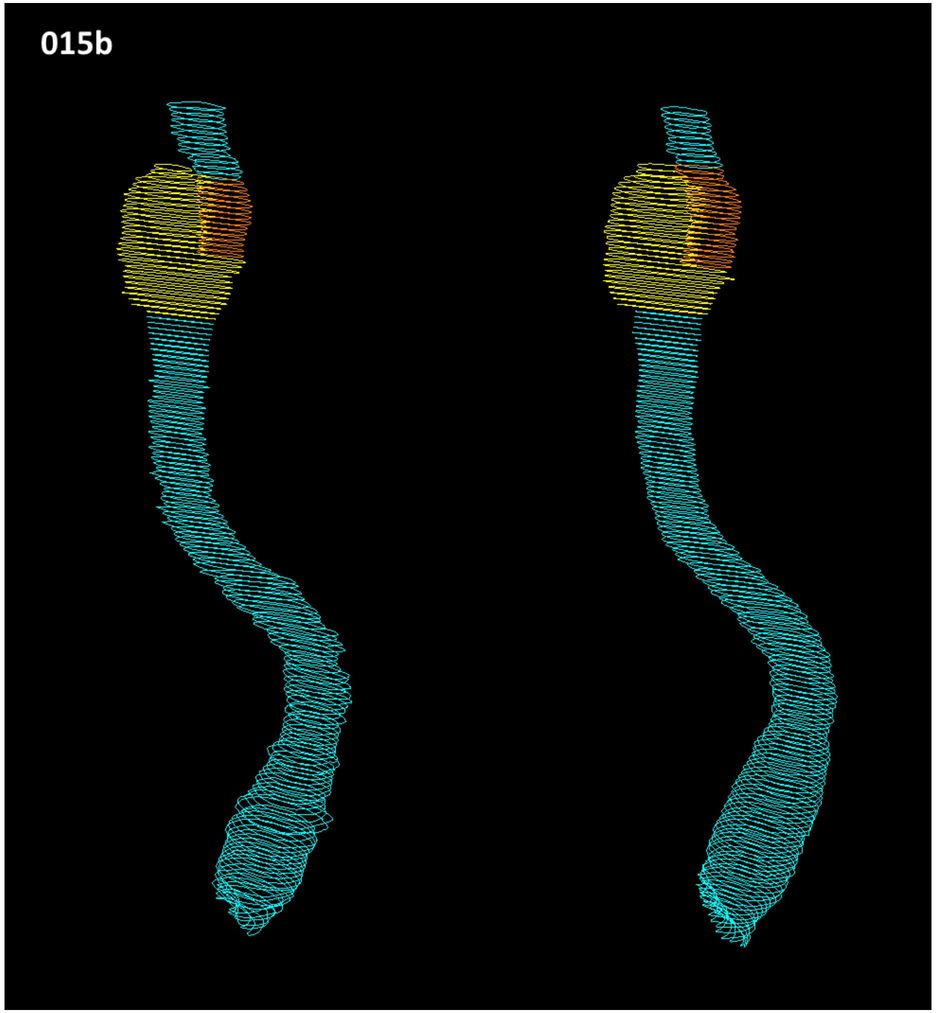

Case 016a

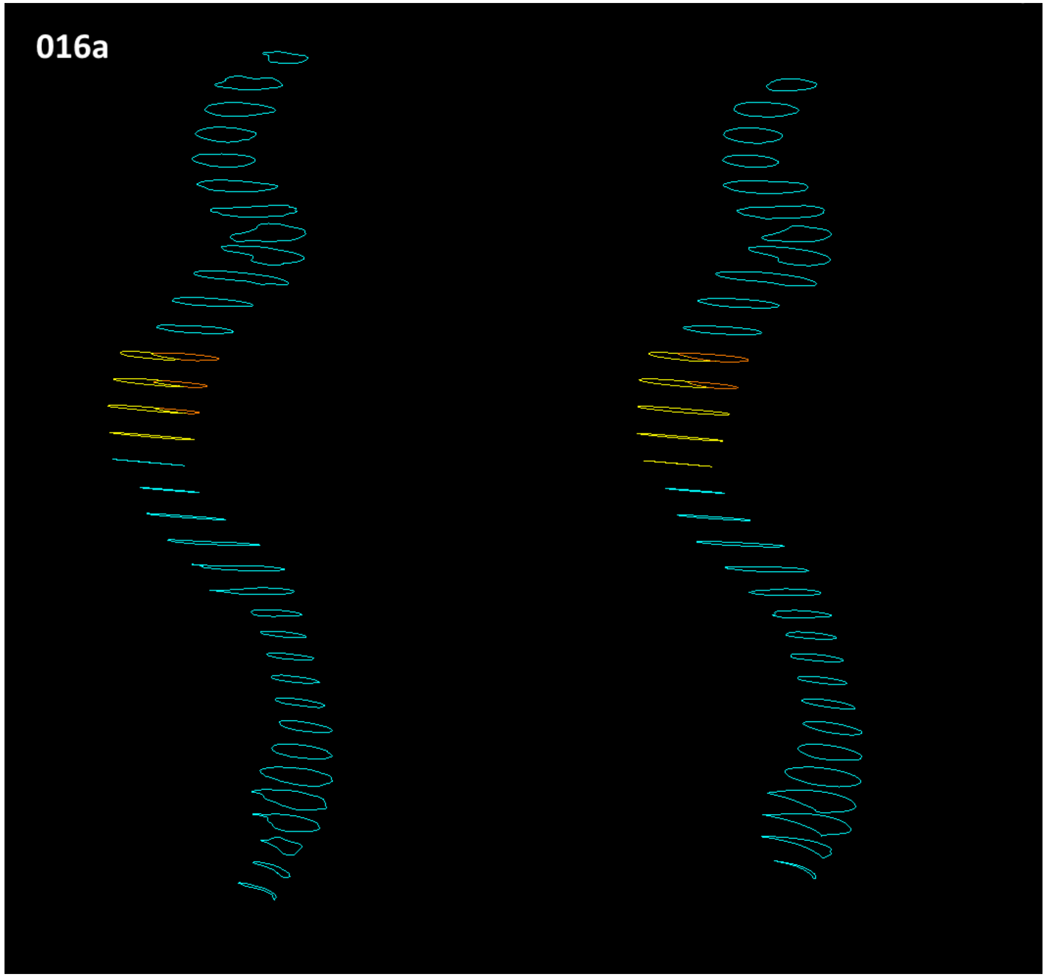

Case 016b

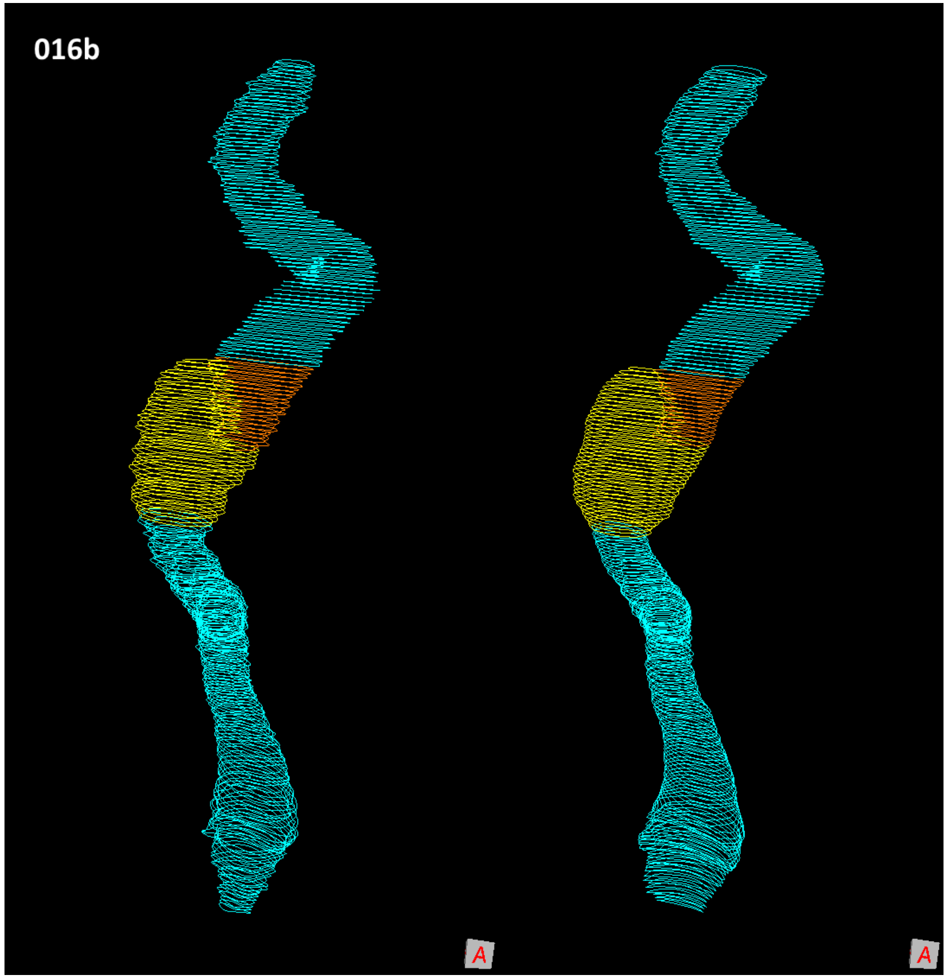

Case 017a

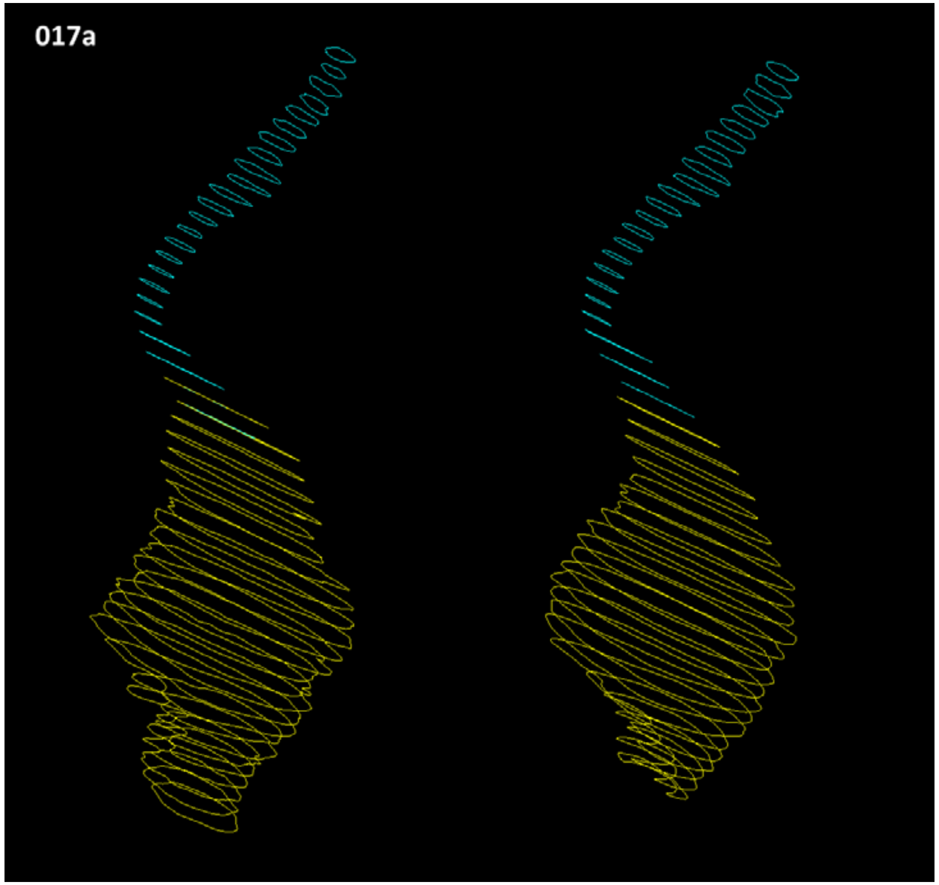

Case 017b

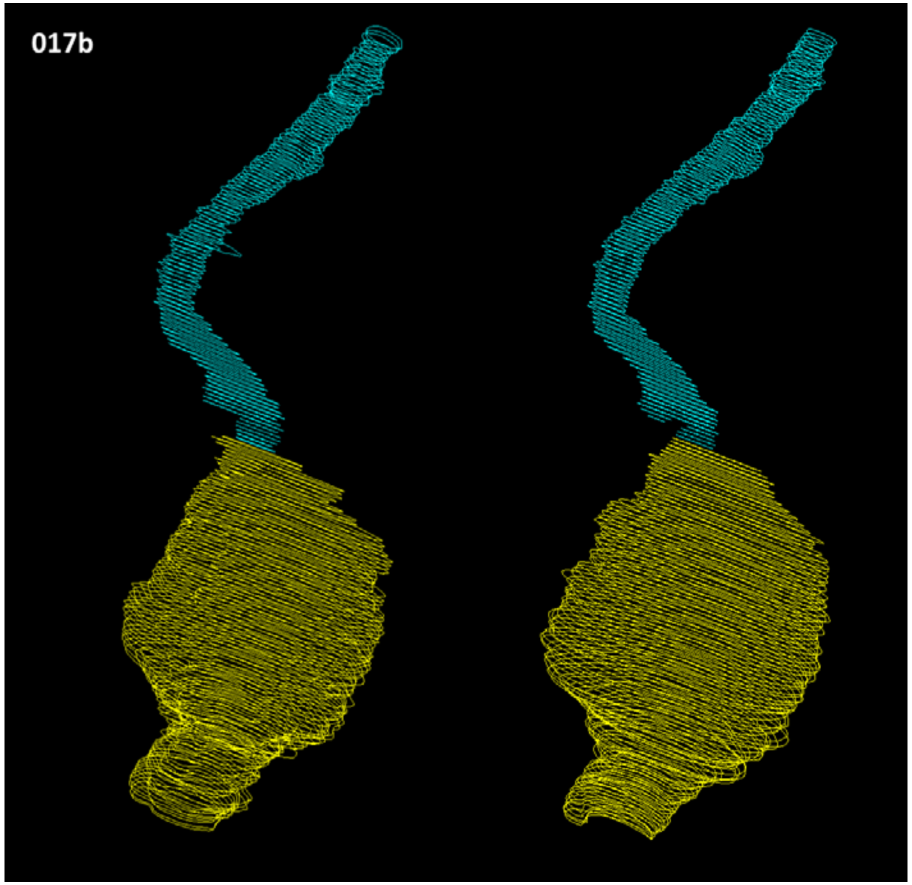

Case 018a

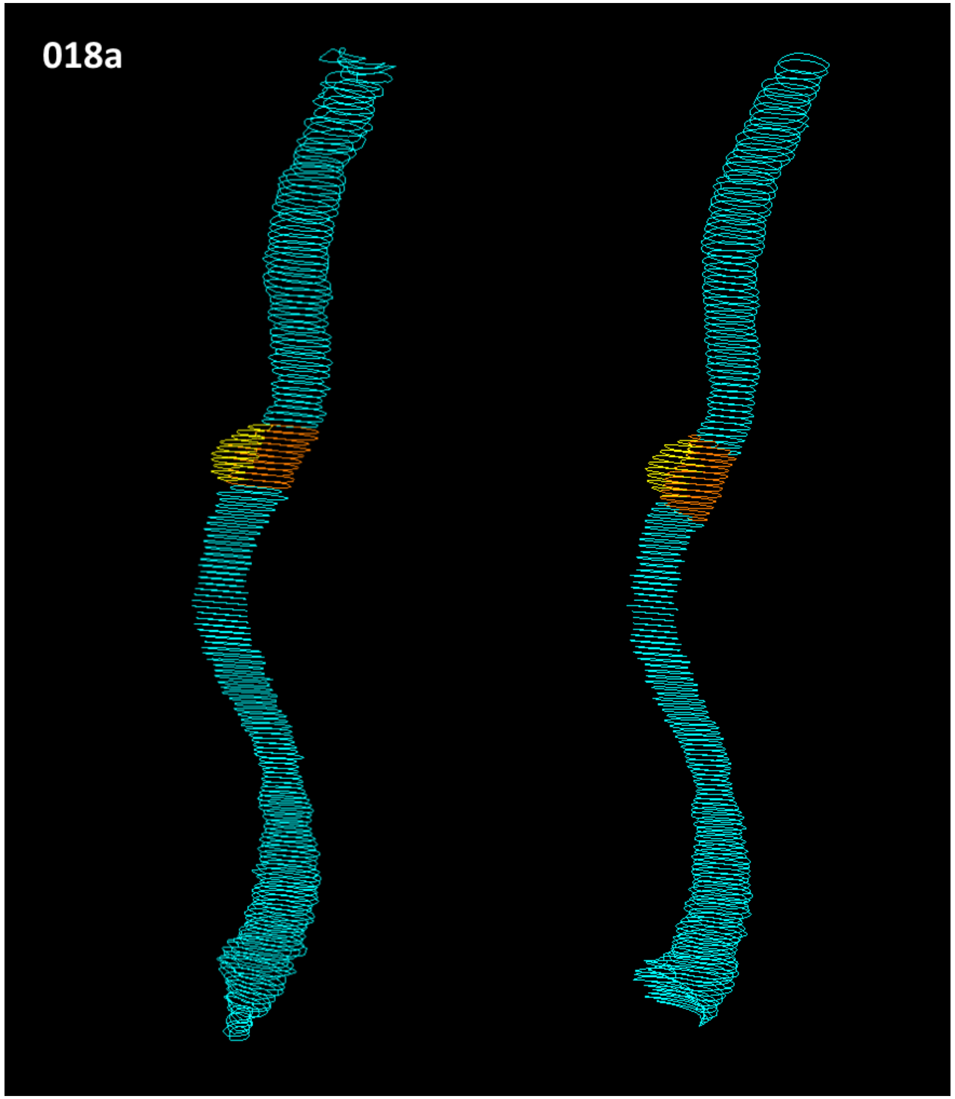

Case 018b

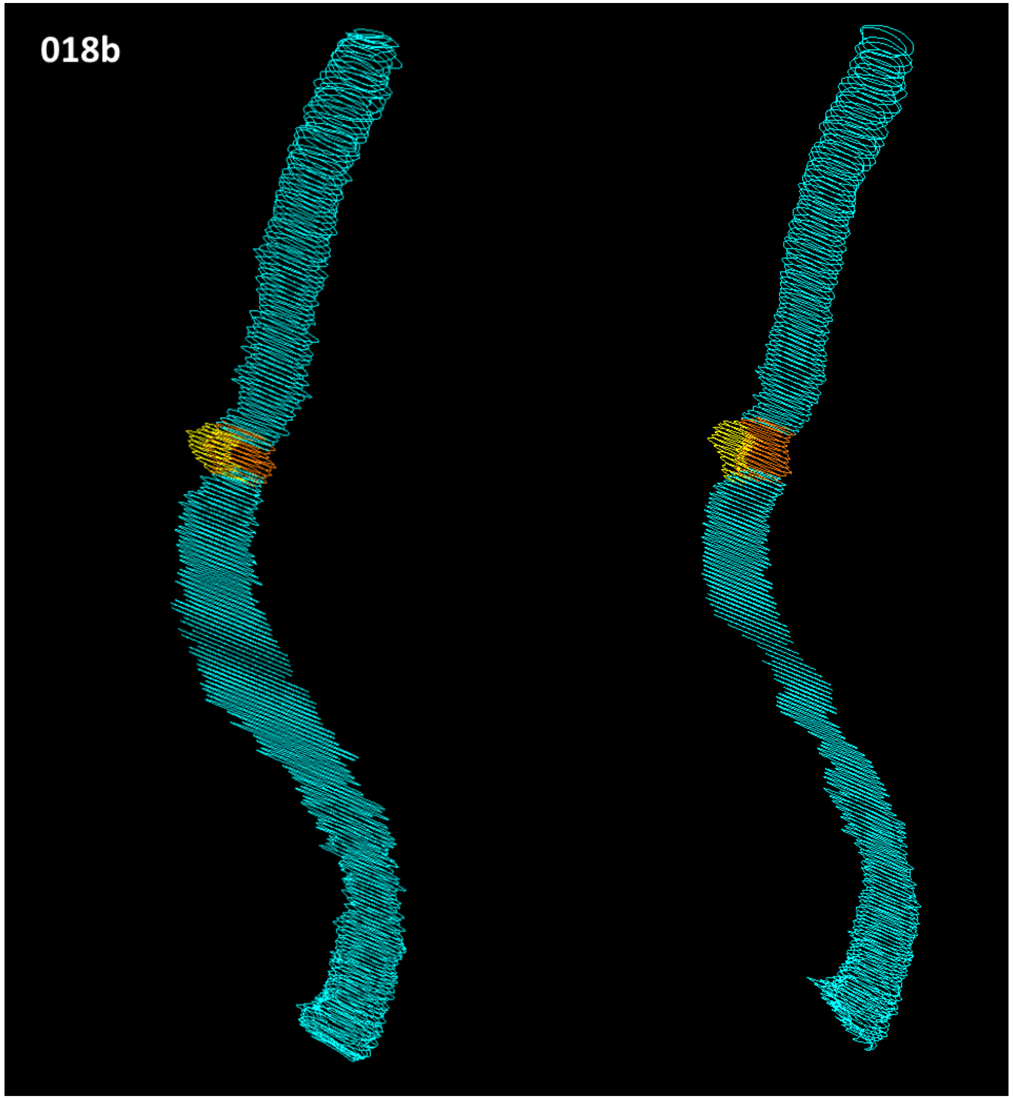

Case 019a

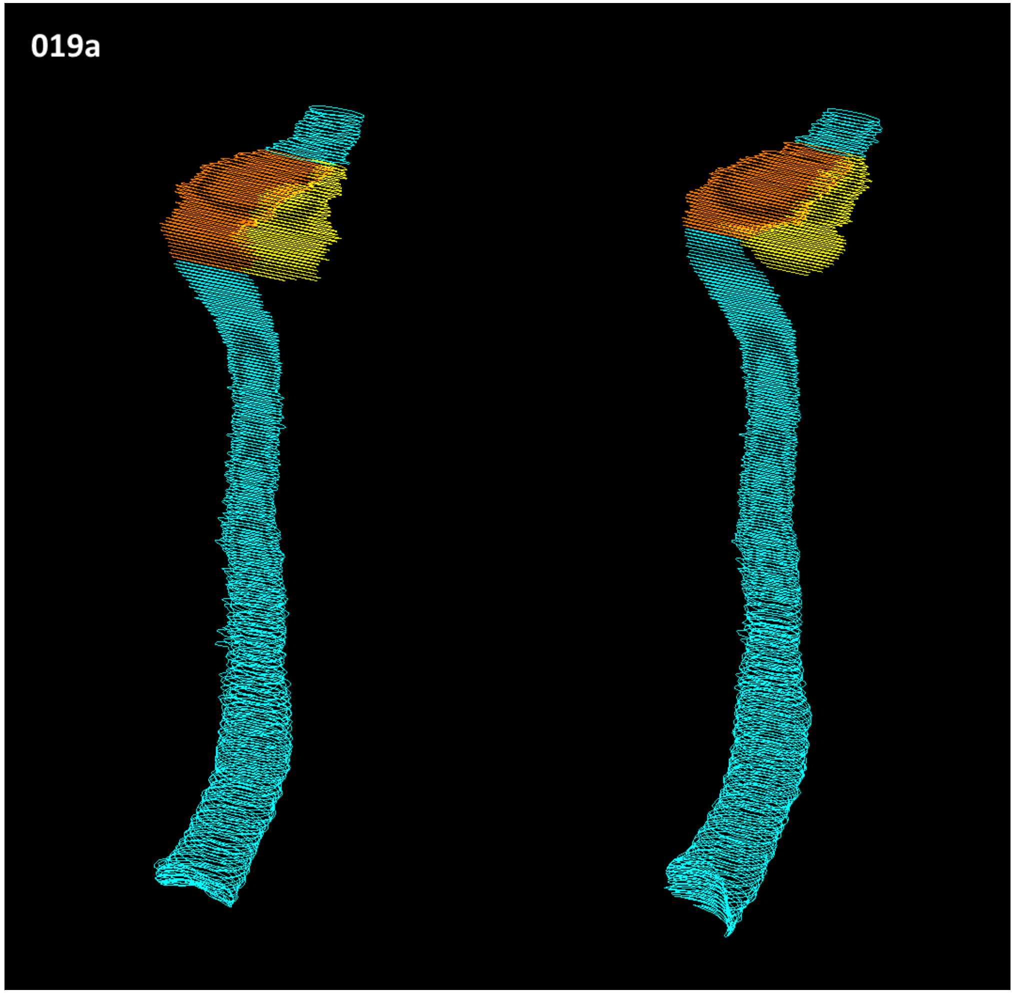

Case 019b

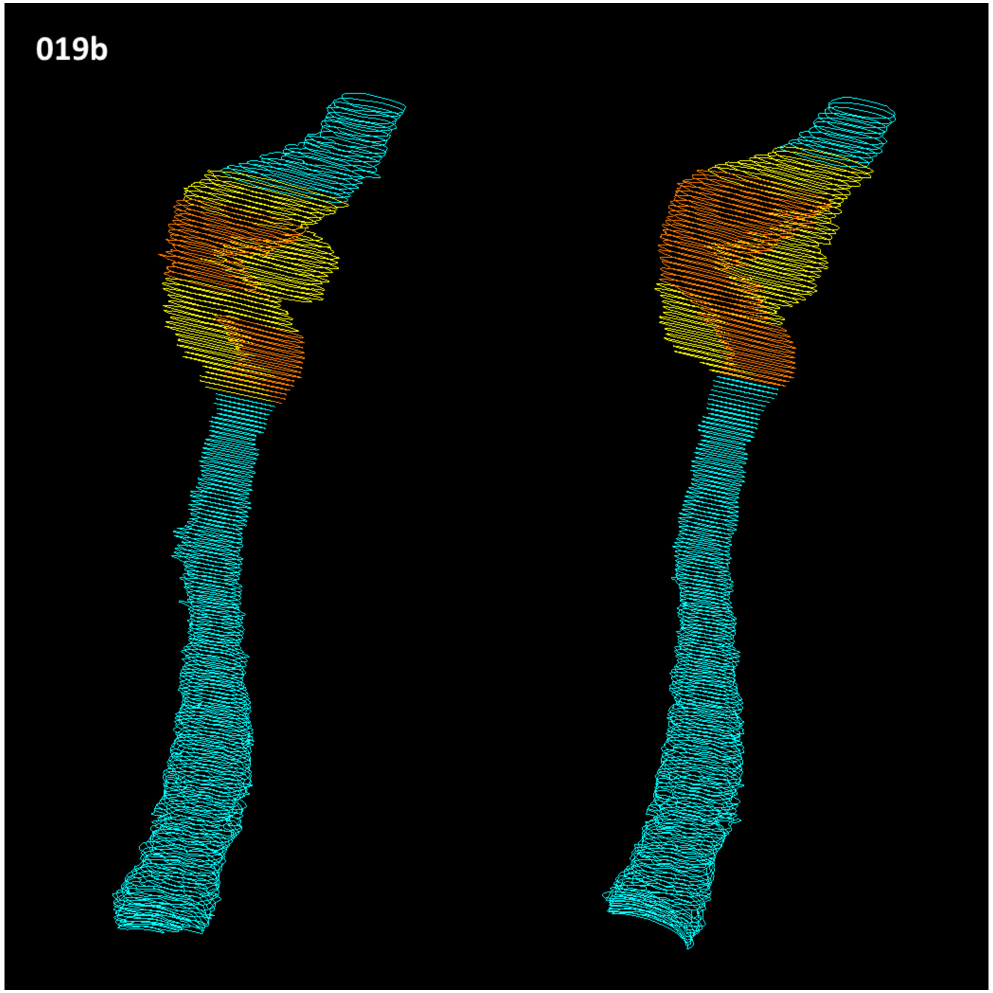

Case 020a

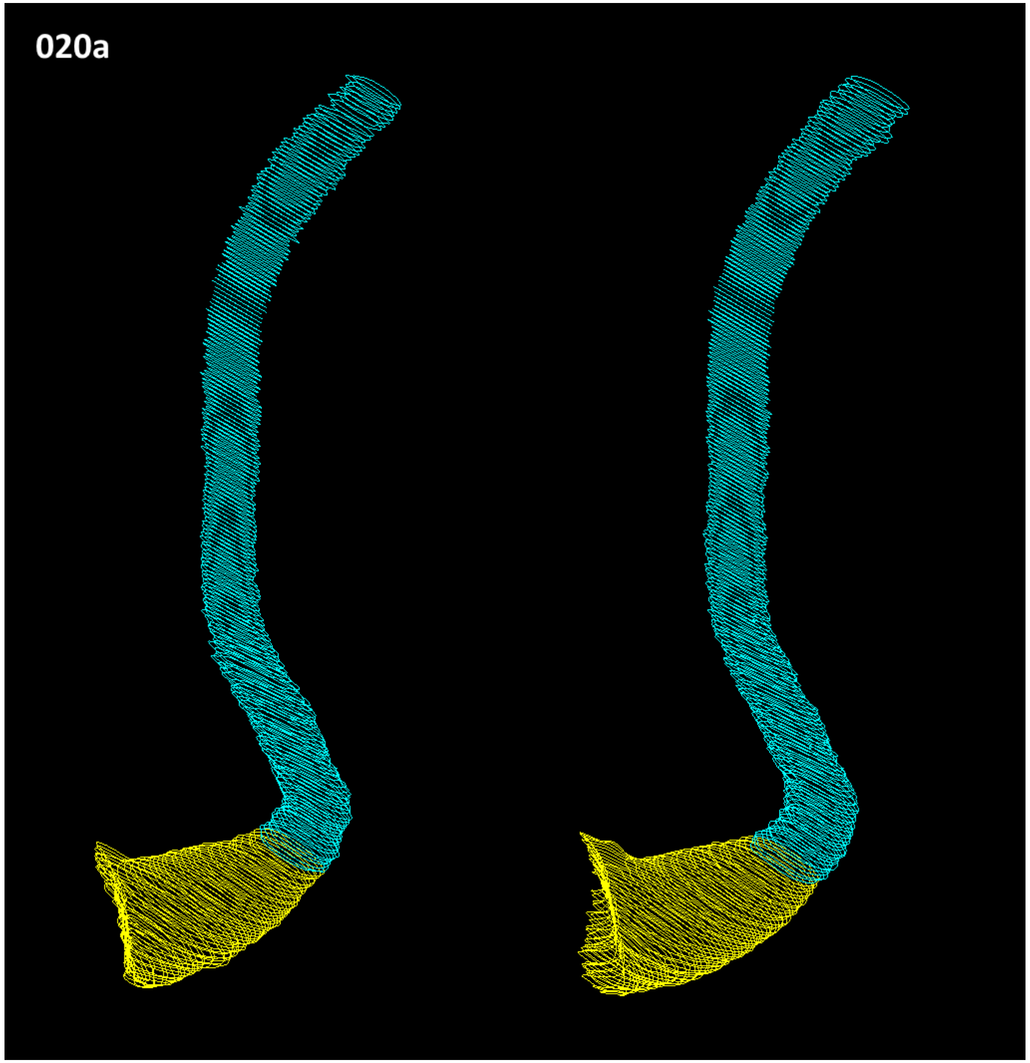

Case 020b

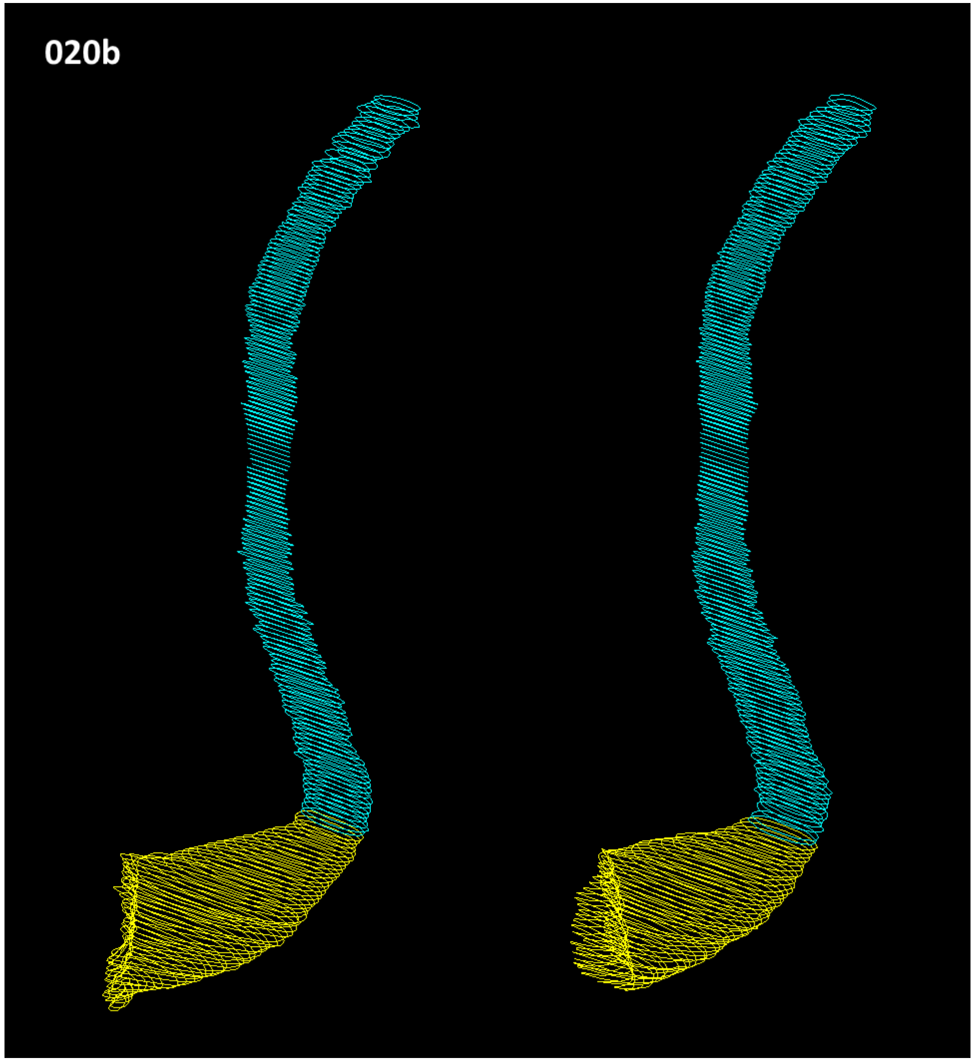

APPENDIX C

Supplemental figure A1-4. Bland-Altman plots showing agreement of two observers on the volume measurement of 40 scans according to the three subfield segmentations, as well as the intraobserver agreement of both observers. The line in the middle represents the mean difference of the volume between the two observers, and the dotted lines represent the upper and lower limits of agreement (mean difference ± 1.96 × standard deviation).

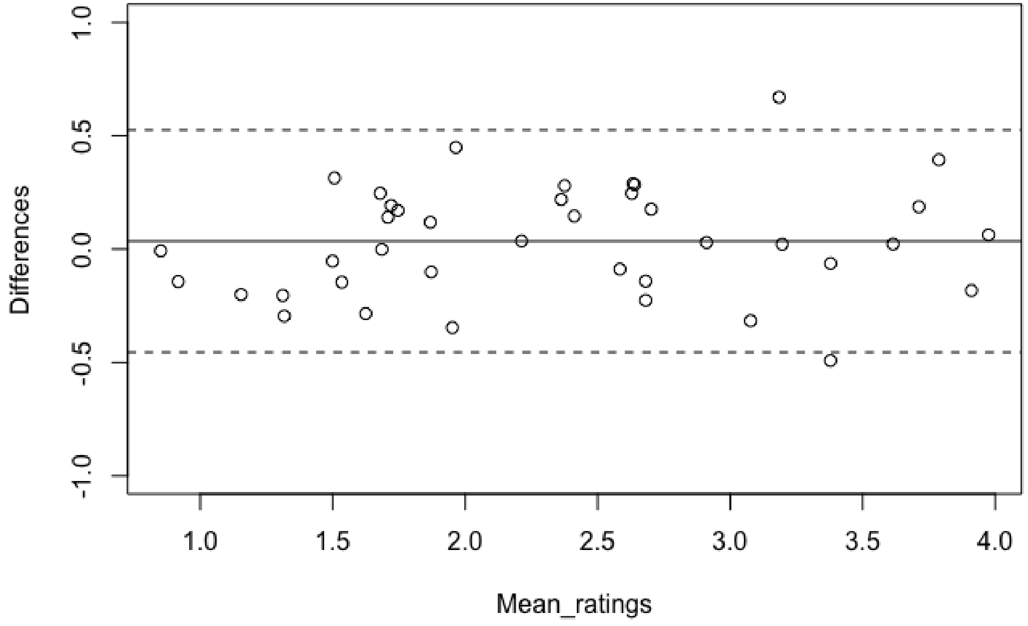

A1. Bland-Altman plot of subfield segmentation "only vessel".

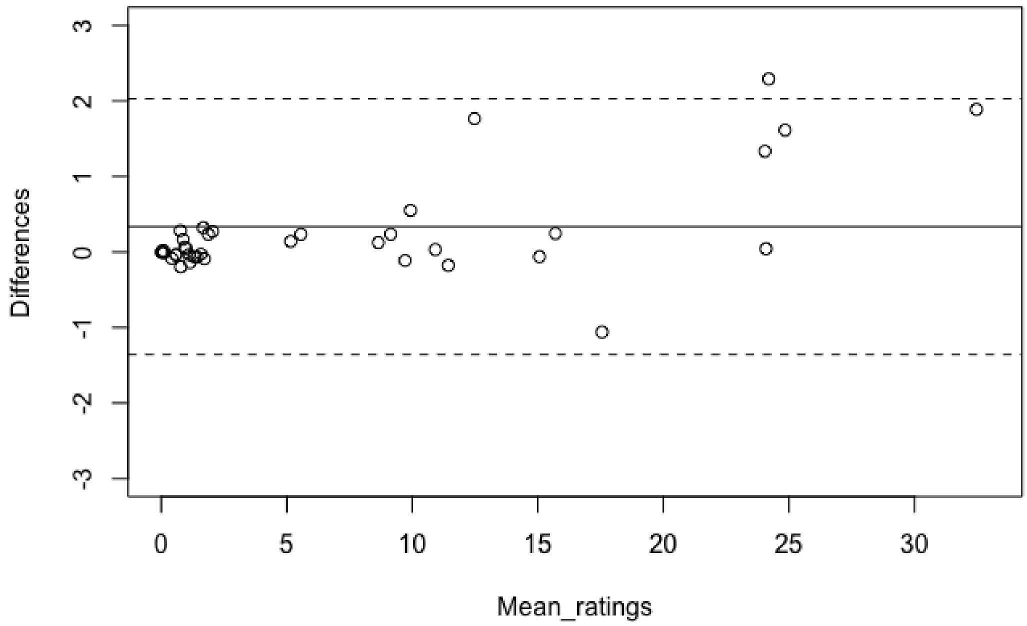

A2. Bland-Altman plot of subfield segmentation "aneurysm sac".

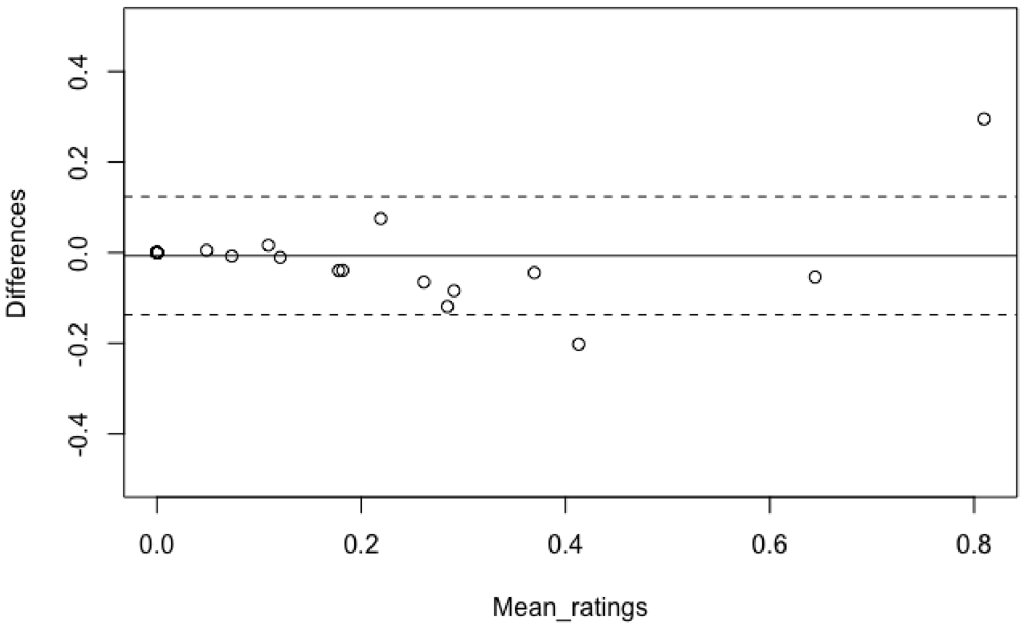

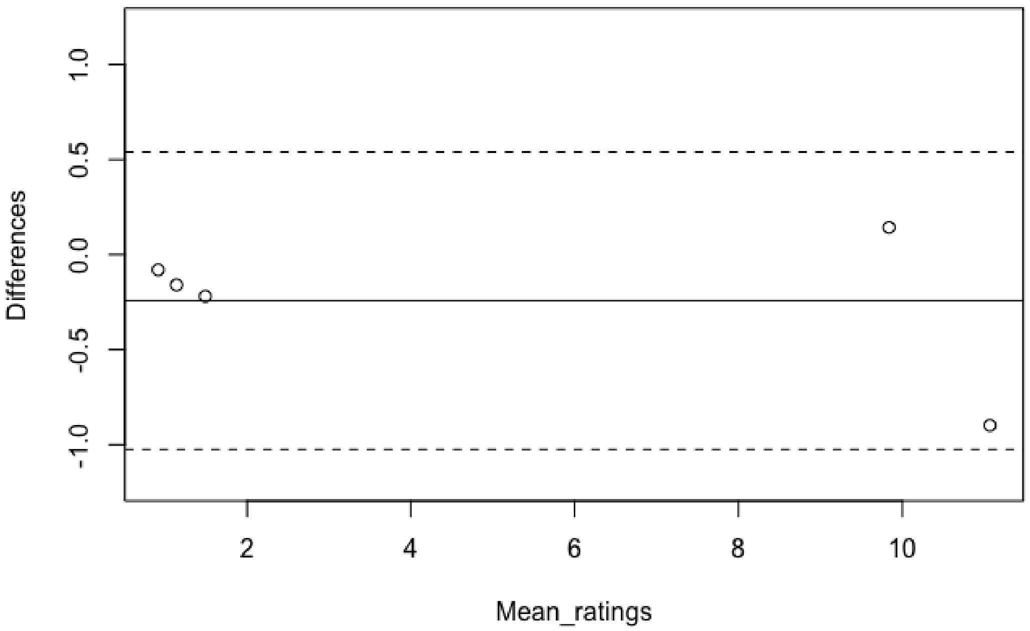

A3. Bland-Altman plot of subfield segmentation “<u>vessel adjacent to aneurysm sac</u>” (top left).

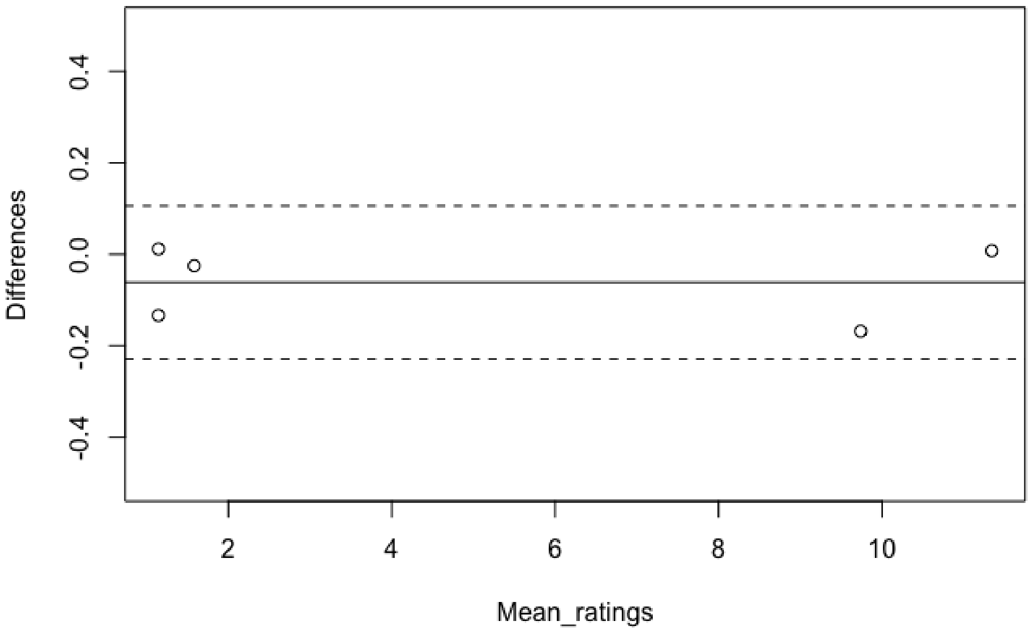

A4. Bland-Altman plots of the <u>intraobserver</u> agreement of measurement <u>method 2</u> for subset of 5 randomly selected cases, for observer 1 (top right) and observer 2 (bottom right).
